# Cadherin 17 Nanobody-Mediated Near-Infrared-II Fluorescence Imaging-Guided Surgery and Immunotoxin Delivery for Colorectal Cancer

**DOI:** 10.34133/bmr.0041

**Published:** 2024-06-21

**Authors:** Youbin Ding, Runhua Zhou, Guangwei Shi, Yuke Jiang, Zhifen Li, Xiaolong Xu, Jingbo Ma, Jingnan Huang, Chunjin Fu, Hongchao Zhou, Huifang Wang, Jiexuan Li, Zhiyu Dong, Qingling Yu, Kexin Jiang, Yehai An, Yawei Liu, Yilei Li, Le Yu, Zhijie Li, Xiaodong Zhang, Jigang Wang

**Affiliations:** ^1^ Department of Medical Imaging, The Third Affiliated Hospital, Southern Medical University (Academy of Orthopedics Guangdong Province), Guangzhou 510515, P. R. China.; ^2^ Shenzhen Clinical Research Centre for Geriatrics and Department of Geriatrics, Shenzhen People’s Hospital; First Affiliated Hospital of Southern University of Science and Technology, Second Clinical Medical College of Jinan University, Shenzhen 518020, Guangdong, P. R. China.; ^3^ NMPA Key Laboratory for Research and Evaluation of Drug Metabolism and Guangdong Provincial Key Laboratory of New Drug Screening and Guangdong-Hongkong-Macao Joint Laboratory for New Drug Screening, School of Pharmaceutical Sciences, Southern Medical University, Guangzhou 510515, P. R. China.; ^4^ Department of Pharmacy, Nanfang Hospital, Southern Medical University, Guangzhou 510515, P. R. China.; ^5^ Department of Neurosurgery and Medical Research Center, Shunde Hospital, Southern Medical University (The First People’s Hospital of Shunde Foshan), Guangzhou 510515, P. R. China.; ^6^ School of Chemistry and Chemical Engineering, Shanxi Datong University, Pingcheng District, Datong, Shanxi Province 037009, P. R. China.; ^7^ State Key Laboratory for Quality Ensurance and Sustainable Use of Dao-di Herbs, Artemisinin Research Center, and Institute of Chinese Materia Medica, China Academy of Chinese Medical Sciences, Beijing 100700, P. R. China.; ^8^ State Key Laboratory of Antiviral Drugs, School of Pharmacy, Henan University, Kaifeng 475004, Henan, P. R. China.; ^9^ Department of Oncology, the Affiliated Hospital of Southwest Medical University, Luzhou 646000, Sichuan, P. R. China.

## Abstract

Surgery and targeted therapy are of equal importance for colorectal cancer (CRC) treatment. However, complete CRC tumor resection remains challenging, and new targeted agents are also needed for efficient CRC treatment. Cadherin 17 (CDH17) is a membrane protein that is highly expressed in CRC and, therefore, is an ideal target for imaging-guided surgery and therapeutics. This study utilizes CDH17 nanobody (E8-Nb) with the near-infrared (NIR) fluorescent dye IRDye800CW to construct a NIR-II fluorescent probe, E8-Nb-IR800CW, and a Pseudomonas exotoxin (PE)-based immunotoxin, E8-Nb-PE38, to evaluate their performance for CRC imaging, imaging-guided precise tumor excision, and antitumor effects. Our results show that E8-Nb-IR800CW efficiently recognizes CDH17 in CRC cells and tumor tissues, produces high-quality NIR-II images for CRC tumors, and enables precise tumor removal guided by NIR-II imaging. Additionally, fluorescent imaging confirms the targeting ability and specificity of the immunotoxin toward CDH17-positive tumors, providing the direct visible evidence for immunotoxin therapy. E8-Nb-PE38 immunotoxin markedly delays the growth of CRC through the induction of apoptosis and immunogenic cell death (ICD) in multiple CRC tumor models. Furthermore, E8-Nb-PE38 combined with 5-FU exerts synergistically antitumor effects and extends survival. This study highlights CDH17 as a promising target for CRC imaging, imaging-guided surgery, and drug delivery. Nanobodies targeting CDH17 hold great potential to construct NIR-II fluorescent probes for surgery navigation, and PE-based toxins fused with CDH17 nanobodies represent a novel therapeutic strategy for CRC treatment. Further investigation is warranted to validate these findings for potential clinical translation.

## Introduction

Colorectal cancer (CRC) is a prevalent gastrointestinal malignancy that accounts for approximately 10% of all diagnosed cancers worldwide, ranking as the second leading cause of cancer-related death [1]. Early symptoms of CRC in patients are often uncharacteristic, and the majority of patients have metastatic cancer when diagnosed. The prognosis for patients with advanced CRC remains poor, with a 5-year survival rate of only 14% [2]. Surgery remains a curative modality for the most patients diagnosed with CRC, especially those with early-stage disease [3]. Clear identification of tumor margins and complete surgical removal of cancer lesions are highly associated with better overall survival and the lower local recurrence of cancer [4]. However, it remains a challenging issue in CRC surgery to identify residual tumor tissues or distinguish the tumor margins during operations.

Visual and tactile distinction alone is insufficient for complete tumor tissue resection. Although conventional imaging techniques, such as computed tomography (CT), magnetic resonance imaging (MRI), ultrasound, and positron emission tomography-CT (PET/CT), can preoperatively aid in determining the precise location, size, and extent of tumors, discerning exact tumor margins or residual tumor tissues can only be achieved through visual and tactile perception during surgery. Therefore, real-time tumor imaging technologies discriminating tumor-positive margins and normal tissues are of great significance for precise tumor resection during surgical procedures [5]. Near-infrared (NIR) fluorescence image-guided surgery is gaining interest due to its ability to visualize tumors intraoperatively [6,7]. NIR-I (400 to 900 nm) and NIR-II (1,000 to 1,700 nm) lights are preferentially applied for image-guided surgery due to their lower photon scattering and less autofluorescence, deeper tissue penetration, and high-resolution imaging compared to visible light [8]. On the other hand, light for NIR-II window is more advantageous than that of NIR-I as it can penetrate deeper tissues (up to 2 cm), produce images with better spatiotemporal resolution, and provide an excellent signal-to-noise ratio [9]. In the past decade, various fluorescent molecules and nanomaterials with NIR-II properties have been largely developed for a broad range of biomedical applications, including imaging for diagnosis and precise intraoperative tumor excision [6,7,10]. Targeted and nontargeted NIR-II probes are 2 categories for imaging-guided surgery navigation [8]. Nontargeted probes without further modification are normally used to visualize sentinel lymph nodes, and they image tumor margins through passive transportation mediated by enhanced permeability retention (EPR) effect [8]. However, these probes lack selectivity and specificity to tumor tissues, leading to inaccurate residual tumor information [11]. In contrast, targeted NIR-II probes are modified by conjugation with agents such as antibodies, peptides, or aptamers that specifically recognize tumor-associated antigens (TAAs) [8]. Targeted NIR-II probes are becoming prevalent for in vivo fluorescence imaging or surgery navigation due to their good selection of tumor tissues, low off-target accumulation in normal tissues, and high sensitivity to tiny tumor lesions, all of which facilitate tumor margin identification and precise resection [11]. In terms of surgery navigation for CRC, several targeted NIR probes have been developed for imaging and imaging-guided surgery, including carcinoembryonic antigen (CEA) antibodies conjugated with fluorophore BM-104 (SGM-101), IRDye800CW (IR800CW) or isotope ^111^In, integrin targeting peptide cRGD [cyclo-(RGDyK)] modified with ZW800-1, VEGFA (vascular endothelial growth factor A) antibody bevacizumab labeled with IR800CW, or pH-activatable probe ONM-100. These probes have shown promising potential for image-guided surgery in various preclinical and clinical settings, but none of them is approved for clinical surgery navigation in CRC patients. This highlights the need for the development of novel targeted NIR-II probes comprising innovative fluorescent agents and new targeting entities beyond conventional antibodies against new TAAs.

Nanobodies (Nbs), small antibodies composed of a single heavy chain variable domain (VHH), are devoid of light chains and constant regions typically found in conventional antibodies. Engineered primarily from camelid heavy-chain only antibodies (hcAbs), Nbs possess high affinity and specificity for antigens despite their smaller size (~15 kDa), which is approximately 1/10th that of full-length immunoglobulin G (IgG) antibodies [12]. The small size of Nbs provides several advantages over conventional full-length antibodies, including enhanced tissue penetration, rapid clearance, easy production and modification, high stability, and reduced immunogenicity.

Selection of TAAs is a determinant factor for the design of targeted NIR-II probes, which can specifically distinguish the tumor margins from surrounding normal tissues. Cadherin 17 (CDH17), the member of cadherin superfamily with 7 extracellular cadherin domains and a short cytoplasm C terminus, has been documented to be overexpressed in tumor tissues of the digestive system, including stomach [13], colorectum [14], liver [15], and pancreas [16]. Recently, high expression of CDH17 protein has also been observed in lung cancers [17]. CDH17 has become a popular target due to its frequent membrane surface expression on cancer cells and minimal expression in normal tissues [14,18]. A variety of therapeutic and imaging modalities targeting CDH17, such as monoclonal antibodies and their conjugates, as well as chimeric antigen receptor T (CAR-T) cell therapy, have been developed and tested in different preclinical settings [19]. It has uncovered that CDH17 is an oncogene for CRC development and could be used as a biomarker for prediction of CRC prognosis and metastasis [20]. Despite the high expression of CDH17 in CRC [21], and its maintenance in metastatic CRC tissues [14], targeting CDH17 for imaging-guided surgery in CRC remains unexplored.

Previously, we identified a Nb that specifically targets CDH17 with high affinity (70.3 nM), termed as E8-Nb, and demonstrated its capacity for NIR-I imaging and toxin delivery in gastric cancer [13]. We found that E8-Nbs can be used to efficiently visualize gastric tumors in vivo and deliver the immunotoxin PE38 (truncated Pseudomonas exotoxin A) in tumors, resulting in an outstanding tumor inhibitory effect [13]. Given that CDH17 is highly expressed in CRC with nearly 100% positivity, and its expression is correlated to poor prognosis [20,21]. We wonder whether E8 Nb conjugated with a NIR-II fluorescent agent could efficiently image CRC tumors and mediate imaging-guided surgical resection in murine CRC models. Meanwhile, immunotoxins targeting TAAs of CRC, such as CEA, EGFR (epidermal growth factor receptor), GPA33 (glycoprotein A33), mesothelin and EpCAM (epithelial cell adhesion molecule) with various toxins including PE38, PE24 or α-sarcin, have achieved promising results in the preclinical and clinical studies [22–25]. Specifically, MOC31PE, composed of EpCAM antibody and the complete Pseudomonas exotoxin A (PE), has shown significant survival extension in patients with metastatic CRC when used as a monotherapy, and has also been shown to provoke antitumor immunity through the induction of ICD However, the potential of toxin delivery with CDH17 targeting in CRC remains to be investigated. Here, we aimed to investigate whether E8-Nb fused with toxin PE38 could effectively suppress CRC tumor development, induce ICD in CRC, and synergistically enhance the antitumor effect of standard chemo-drugs such as 5-fluorouracil (5-FU).

In this study, we conjugated the E8-Nb with the NIR fluorescent dye IR800CW, which possesses properties of both NIR-I and NIR-II windows [26], to design a targeted NIR-II fluorescent probe, E8-Nb-IR800CW (E8-IR800CW). We used this probe to visualize CRC tumors and perform imaging-guided surgery in murine CRC tumor models. Additionally, the inhibitory effect of E8-Nb-PE38 (E8-PE38) immunotoxin on CRC cells was also assessed in vitro and in vivo. Our results demonstrate that the NIR-II imaging probe E8-IR800CW effectively images subcutaneous tumors of CRC expressing CDH17 in nude mice and allows for imaging-guided precise tumor removal. The E8-PE38 immunotoxin significantly suppresses CRC tumor growth through the induction of cancer cell apoptosis and ICD and extends survival in murine CRC tumor models. The addition of E8-PE38 immunotoxin to the standard drug regimen 5-FU can synergistically augment the antitumor performance and further prolong survival. Together, these results indicate that CDH17 can serve as a potential target for NIR-II imaging-guided surgery and immunotoxin therapy in CRC, warranting further translational assessment in clinical settings.

## Materials and Methods

### Cell lines and cell culture

Four CRC cell lines (HCT116, HT115, T84, and LOVO) and 4T1 cells were procured from the American Type Culture Collection (Manassas, VA). T84 cells were cultured in Dulbecco’s modified Eagle’s medium/F12 (Gibco, USA) containing 10% fetal bovine serum (ExCell Bio, China) and 1% penicillin/streptomycin, while HCT116, HT115, 4T1, and LOVO cells were cultured in RPMI 1640 (Gibco, USA) containing 10% fetal bovine serum and 1% penicillin/streptomycin. The cells were incubated in a humidified atmosphere with 5% CO_2_ at 37°C.

### Fluorescent probe synthesis and characterization

IR800CW maleimide (LI-COR, USA) was mixed with the Nbs (1 mg/ml, pH 8.0) at a molar ratio of 2:1 and allowed to react for 2 h at room temperature (RT) in the dark. The unbound dye was removed through a 3K Amicon Ultra-15 Centrifugal Filter (Millipore). Ultraviolet-visible-NIR I (UV-vis-NIR I) absorption and emission spectra of the probe were measured using a Spark Multimode Microplate Reader (Spark, Switzerland), while the concentration of labeled Nb was determined by the bicinchoninic acid (BCA) protein assay kit (Thermo Fisher Scientific, USA). The NIR-II emission spectra of IR800CW were measured on FLS980 (Edinburgh Instruments, UK). Sodium dodecyl sulfate–polyacrylamide gel electrophoresis (SDS-PAGE) analysis was used to visualize the dye-labeled proteins detected with Sapphire FL Biomolecular Imager (Azure Biosystems, USA).

### Cell knockdown of CDH17

The specific details of the cellular knockdown of CDH17 were carried out as described previously [13]. Short hairpin RNA (shRNA) sequences used in this study: CDH17 shRNA#3: 5′-CCAGTCCCTATCACCATAGAA-3′, control shRNA: 5′-GCAAGCTGACCCTGAAGTTTA-3′.

### Competition assay for E8-IR800CW specificity

To determine the specificity of E8-IR800CW to CDH17 protein, free E8-Nb and recombinant CDH17 protein (domain 1–3) were used to compete the binding activity of E8-IR800CW through cell enzyme-linked immunosorbent assay (ELISA) assay. For the competition with free E8, cells were incubated with excessive unlabeled E8 Nb for 1 h. Subsequently, E8-IR800CW was added to the cells and further incubated for 1 h. For the competition with free CDH17, E8-IR800CW cells were first incubated with free CDH17 protein for 1 h. The mixed solution was then added to the cells and further incubated for 1 h. Cells were washed 3 times with phosphate-buffered saline (PBS), and the fluorescence intensity was measured by the Sapphire system.

### Colony formation

Cells were seeded into 6-well plates at a density of 1 × 10^3^ cells per well. After incubation overnight, E8-PE38 and Con-PE38 were appropriately diluted in 2 ml of medium and incubated with cells. After 1 week, the cells were washed with PBS and fixed with 4% paraformaldehyde for 1 h at RT. Subsequently, colonies were stained using 0.1% crystal. The clone number was quantitatively analyzed by ImageJ software.

### Mass spectrometry analysis

Cells treated with E8-PE38 or PBS were collected and lysed with RIPA lysis buffer containing protease inhibitors. The protein concentrations of the samples were determined using a BCA assay kit. Next, the samples were subjected to reduction and alkylation with 5 mM dithiothreitol and 20 mM indole acetic acid for 30 min. Proteins were finally digested and then analyzed by liquid chromatography–tandem mass spectrometry (LC-MS/MS) (Thermo Scientific, USA). By the abundance signals of the PBS or E8-PE38 group, differentially expressed proteins (DEPs) were identified based on the criteria of fold changes > 1.5 and *P* < 0.05.

### NIR-II fluorescence in vivo imaging

E8-IR800CW and Con-Nb-IR800CW (Con-IR800CW) were intravenously injected into tumor-bearing mice at a dose of 100 μg per mouse. To acquire NIR-II imaging, the whole-body imaging was captured at 4, 8, 12, and 24 h with a NIR-II in vivo imaging system (MARS, China) after drug administration. The excitation laser had a wavelength of 808 nm and a power setting of 3,000 mA. The exposure time was 100 ms, with a light filter of 900-nm long-pass filters fixed in front of the lens. At 24 h, tumors with high fluorescence intensity were resected under the fluorescence real-time dynamic imaging and further observed for complete resection.

### NIR-I fluorescence in vivo imaging

To assess the distribution of immunotoxin in mice, E8-PE38Mut or Con-PE38Mut conjugated with IR800CW was intravenously injected into tumor-bearing mice (200 μg per mouse). The fluorescence enrichment was recorded at 4 time points (3, 8, 12, and 24 h) using the IVIS Spectrum imaging system (PerkinElmer, USA) under an excitation wavelength of 745 nm and an emission wavelength of 840 nm. At 24 h, major organs (heart, liver, spleen, lung, kidney) and tumors were dissected for ex vivo fluorescence imaging.

### Mouse xenograft models and treatment

Animal experiments were reviewed and approved by the Animal Welfare and Ethics Committee of Shenzhen Peopleℳs Hospital. All nude mice were provided by GEMPHARMATECH of Guangdong Province. To establish the subcutaneous tumor models, 100 μl of PBS containing approximately 3 × 10^6^ HCT116 cells, 3 × 10^6^ HT115 cells, or 5 × 10^6^ T84 cells was injected into the right lower back of the mice. When the tumor volume reached approximately 100 mm^3^, E8-PE38 or 5-FU was administered to tumor-bearing mice every other day for 7 injections. Antitumor performance was assessed with different doses of E8-PE38 through intravenous injection in HCT116 (0.6 and 0.8 mg/kg), HT115 (0.4 mg/kg), and T84 (0.6 mg/kg) tumor models. For combination therapy, 25 mg/kg 5-FU was intraperitoneally injected into HCT116-tumor bearing mice every other day. During treatment, the tumor size was recorded by measuring the width and length of the tumor daily, and tumor volume was calculated according to the following formula: tumor length × width^2^/2. Mice were euthanized when they showed obvious signs of discomfort or when tumor volumes reached 2,000 mm^3^. For the survival study, the mice were normally euthanized when the tumor reached a size of 2,000 mm^3^ according to the requirements of the Animal Welfare and Ethics Committee_._

### Statistical analysis

All the data are presented as the mean ± SEM, unless otherwise specified. Statistical analysis was performed using GraphPad Prism software (GraphPad, USA). Differences between the 2 groups were analyzed using a two-tailed *t* test. Tumor weight and various toxicological parameters among the 4 groups were analyzed using one-way analysis of variance (ANOVA). In vitro, cell viability and tumor growth curves were evaluated with two-way ANOVA. Survival curves between groups were compared through the application of the log-rank test. *P* < 0.05 was considered significantly different, and any significant differences were expressed using asterisks (*P < 0.05, **P < 0.01, ***P < 0.001, ****P < 0.0001).

## Results

### In vitro and in vivo validation of high CDH17 expression in CRC

**Fig. 1.**
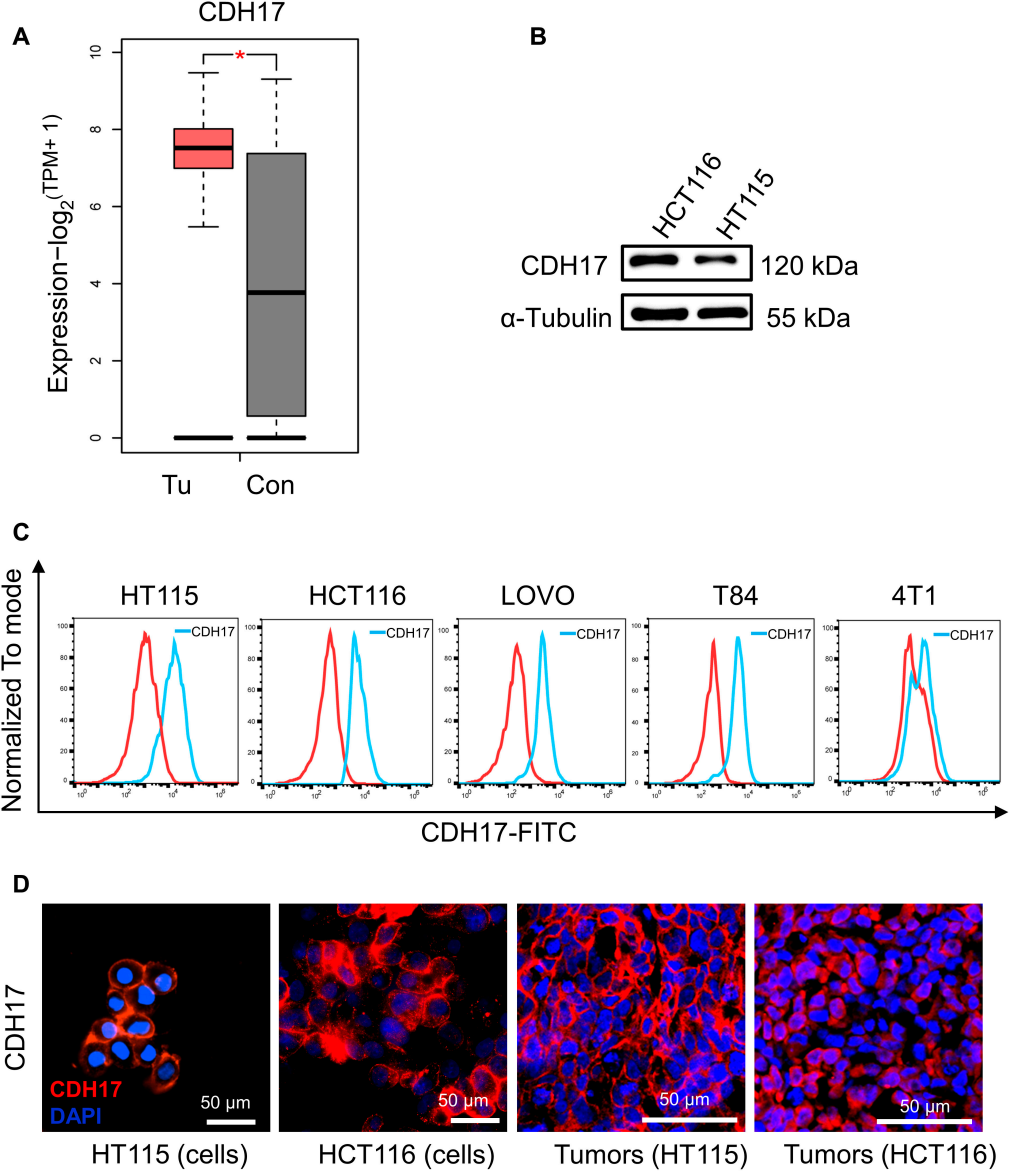
Recapitulation of CDH17 expression in CRC. (A) CDH17 RNA expression (TCGA/GTEx) in colon cancers and normal colon tissues. *n* = 275 (tumors), *n* = 349 (controls). (B) CDH17 expression in CRC cell lines (HCT116 and HT115) was determined by Western blot. (C) CDH17 membrane expression in CRC cell lines analyzed by flow cytometry. (D) CDH17 membrane expression in CRC cell lines (HT115 and HCT116) and subcutaneous tumor tissues induced by HT115 and HCT116 determined by immunofluorescence staining. Scale bars, 50 μm.

CDH17 expression has been demonstrated in CRC human samples and can be used along with other markers, such as mucin 2 (MUC2) and cyclooxygenase-2 (COX-2), to predict disease progression and prognosis in CRC patients [18,21]. To further confirm the expression of CDH17 in CRC, the RNA expression of CDH17 in colon adenocarcinoma (COAD) tissues was examined using data from The Cancer Genome Atlas (TCGA) and Genotype Tissue Expression (GTEx) databases. Results indicated significant up-regulation of CDH17 mRNA in COAD tissues compared to normal colon tissues (Fig. 1A). The expression of CDH17 in CRC cell lines HCT116 and HT115 was also confirmed by Western blotting (Fig. 1B). Cell membrane expression of CDH17 was further verified with flow cytometry in 4 CRC cell lines (HT115, HCT116, LOVO, and T84) and a negative control cell line (4T1). All 4 CRC cell lines except 4T1 cells showed the expression of CDH17 in membrane with different levels (Fig. 1C). Immunostaining in CRC cell lines (HT115 and HCT116) and CRC xenograft tissues induced with the same cell lines further confirmed the membrane expression of CDH17 on tumor cells (Fig. 1D). Together, these results demonstrate that CDH17 is up-regulated in CRC cells and tumor tissues and could be used as a membrane protein marker for the development of targeted imaging and therapy against CRC.

### In vitro characterization of CDH17 Nb-based NIR-II probe E8-IR800CW

**Fig. 2.**
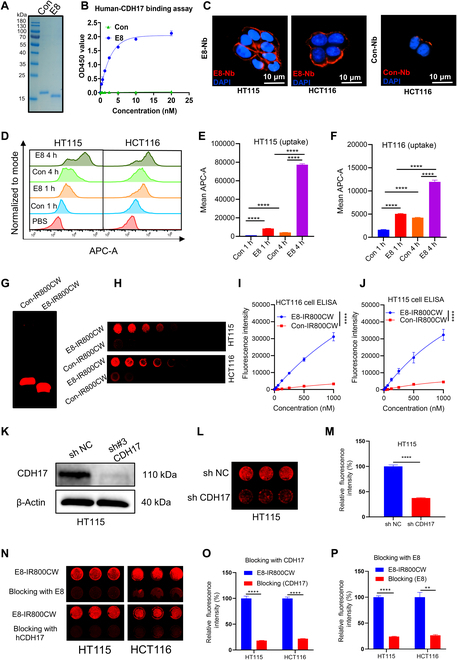
In vitro assessment of E8-IR800CW probe in CRC cells. (A) SDS-PAGE analysis of purified CDH17 Nb E8 and control Nb (Con). (B) Binding capacity of E8 and Con Nbs to CDH17 protein determined by ELISA. (C) Immunofluorescence cell staining with E8 and Con Nbs for CRC cell lines; Nb signal (red) was amplified with the antibody against HA integrated into the Nb sequence. Scale bars, 10 μm. (D) Flow cytometry uptake detection for E8 and Con Nb at 2 time points (1 and 4 h). (E and F) Quantification of the cellular uptake of the E8 and Con Nbs (*n* = 3) in HT115 and HCT116 cells. (G) Fluorescent SDS-PAGE analysis of E8-IR800CW and Con-IR800CW. (H) Binding activity of E8-IR800CW and Con-IR800CW in CRC cell lines detected by cell ELISA (*n* = 3). (I and J) Quantification of the binding activity of E8-IR800CW and Con-IR800CW in CDH17-expressing HT115 and HCT116 cell lines. (K) Knockdown of CDH17 in HT115 cells with shRNA determined by Western blot. (L) Specificity of E8-IR800CW to CDH17 in CDH17-knockdown HT115 cells determined by ELISA. (M) Quantification of cell ELISA for (L) (*n* = 3). (N) Competition assay of E8-IR800CW with free E8 Nb and free hCDH17 domain 1–3 determined by cell ELISA. (O and P) Quantification for E8-IR800CW competition assay with the free E8 Nb and free CDH17 domain 1–3 for (N). *P < 0.05, **P < 0.01, and ***P < 0.001.

First, the Nb E8 against human CDH17 domain 1–3 and an irrelevant Nb control (Con) were purified as previously described [13] and examined with SDS-PAGE gels, showing 2 protein bands with high purity within the expected range of 15 to 17 kDa (Fig. 2A). The binding affinity for E8 Nb for human CDH17 protein was previously determined to be 74 nM [13]. ELISA assay confirmed the binding activity of the E8 Nb against human CDH17 protein (domain 1–3), exhibiting a higher binding capacity compared to the irrelevant control (Con) Nb (Fig. 2B). The CDH17-positive CRC cell lines HT115 and HCT116 could be effectively stained by E8 Nb and showed clear membrane staining, while the cells stained by Con Nb showed weak signals (Fig. 2C). Effective uptake of imaging probes by cancer cells is crucial to obtain stable and sustainable in vivo tumor imaging. Hence, uptake of Nb E8 by CRC cells was assessed by flow cytometry, and the data showed that the fluorescent signal in cells treated with Cy5-labeled E8 Nb was significantly increased over incubation time when compared with the Con Nb-Cy5 in both CRC cell lines HT115 and HCT116 (Fig. 2D to F). Specifically, the fluorescence intensity for E8-Cy5 was approximately 20-fold greater than that of the Con Nb-Cy5 in HT115 cells after 4 h incubation (Fig. 2E). Next, NIR-II fluorescent probes were synthesized by conjugating IR800CW to Nbs (E8 and Con) through the reaction of C-terminal cysteines of the Nbs with maleimide in IR800CW molecules (Fig. S1A). First, the results of the E8-IR800CW probe were determined by SDS-PAGE gel (Fig. 2G). Subsequently, the absorption and emission spectra of E8- IR800CW were measured. The UV-vis spectrum analysis showed that E8-IR800CW exhibited the peak of the absorption spectrum at 778 nm, which was similar to free IR800CW (774 nm), and the peak of the emission spectrum at NIR-I window was at 789 nm (Fig. S1C and F). The UV absorption spectrum of E8-IR800CW at different concentrations was similar in shape (Fig. S1D). The emission spectrum of E8-IR800CW for the NIR-II signal was detectable from 1,000 to 1,300 nm in our system (Fig. S1G). In addition, the fluorescence intensity of E8-IR800CW was linearly and positively correlated with the concentration of E8-IR800CW in the tested concentration range (0 to 15 μg/ml) (*R*^2^ = 0.9999) (Fig. S1E). Meanwhile, the E8-IR800CW probe could be clearly observed the NIR-II signal in a concentration-dependent manner under the excitation of NIR-II system MARS, demonstrating its suitability for NIR-II imaging (Fig. S2A and B). After conjugation, an ELISA assay was conducted to examine whether IR800CW affects the binding ability of the Nbs against the CDH17 protein. As shown in Fig. S1B, the E8-IR800CW probe maintained the comparable binding activity to the free E8 Nb, and no binding activity was detected for either the free Con Nb or Con-IR800CW, indicating that IR800CW does not alter the activity of E8 Nb. The biosafety of both fluorescent probes was evaluated in vitro, and any cytotoxic effect on cancer cells (HT115 and HCT116) for both probes was not found even at high concentrations (Fig. S3). Next, cell ELISA was utilized to further verify the binding ability of E8-IR800CW to CRC cells (HT115 and HCT116) (Fig. 2H). The fluorescence intensity of E8-IR800CW showed a concentration-dependent increase in both CRC cell lines, but Con-IR800CW did not suggest any binding activity (Fig. 2I and J), suggesting that the E8-IR800CW probe can effectively recognize CDH17 on the cell membrane with the strong binding ability. To confirm the specificity of E8-IR800CW against CDH17, we knocked down CDH17 protein in HT115 with CDH17 shRNA lentivirus (Fig. 2K). Cell ELISA data uncovered that knockdown of CDH17 protein in HT115 cells resulted in a significant reduction in the fluorescence intensity of E8-IR800CW, further confirming its specificity for CDH17 (Fig. 2L and M). Finally, the specificity of E8-IR800CW against CDH17 was also confirmed with the competition assay with free E8 Nb and free hCDH17 protein in HT115 and HCT116 cells. Both competition experiments showed that either free E8 Nb or hCDH17 protein could effectively block the binding of E8-IR800CW to the CRC cells highly expressing CDH17 (Fig. 2N to P). Collectively, these findings demonstrate that the E8-IR800CW probe can specifically and efficiently recognize CDH17 protein and CRC cell lines overexpressing CDH17 and can be rapidly internalized by CDH17-expressing CRC cells. These results support the potential of the E8 Nb for NIR-II imaging and targeted therapy against CRC.

### E8-IR800CW effectively images CRC tumors in NIR-II window and directs fluorescence-guided surgery

**Fig. 3.**
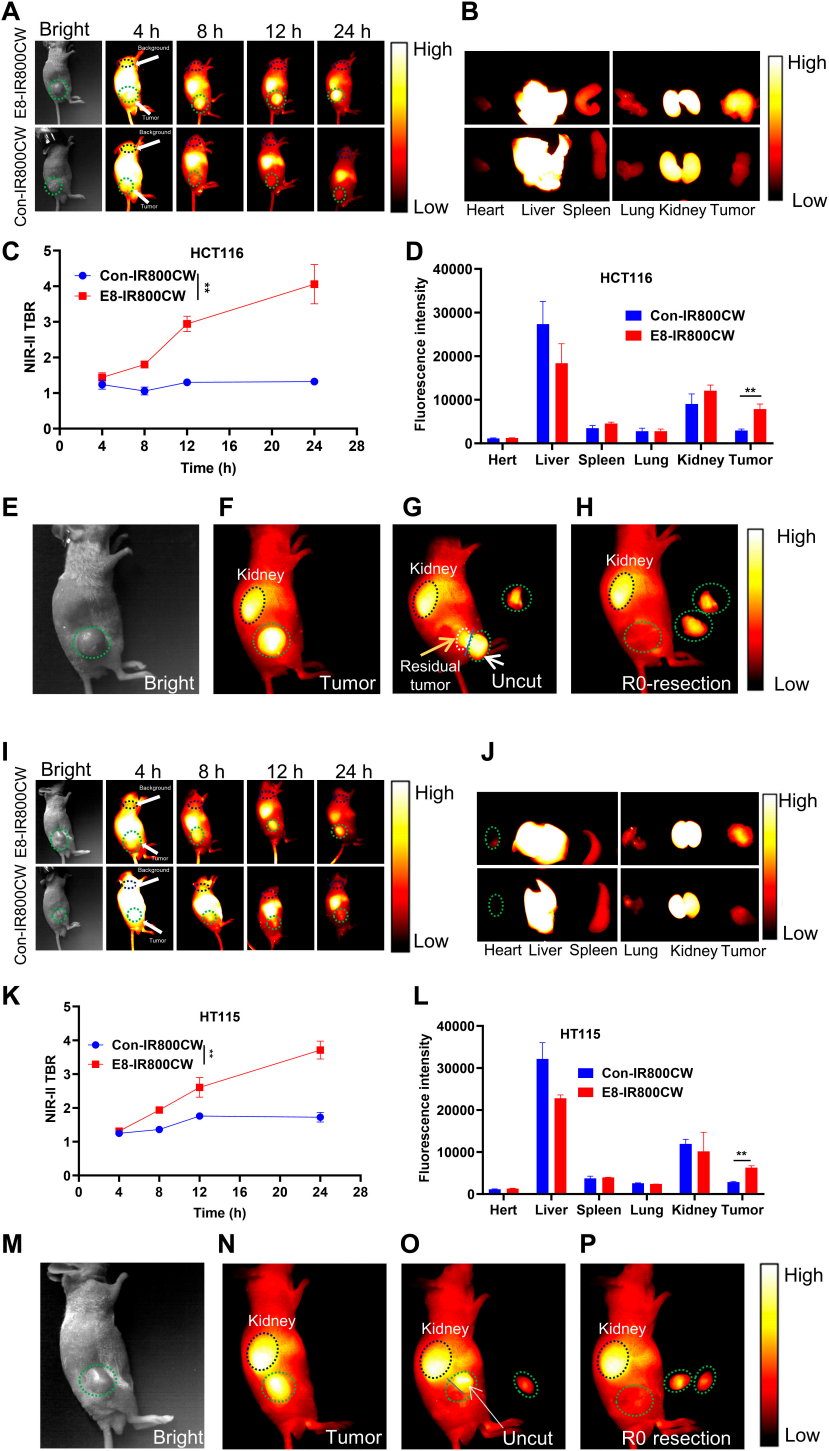
In vivo NIR-II imaging and fluorescence-guided precise tumor resection with E8-IR800CW in CRC murine models. (A) Whole-body imaging for HCT116 tumor-bearing mice under different time points (4, 8, 12, and 24 h) after intravenous injection with E8-IR800CW or Con-IR800CW (100 μg per mouse). The black cycles indicated the skin background, and the green cycles enclosed the tumors. (B) Ex vivo imaging of major organs dissected from in vivo-imaged mice from (A) at 24 h (*n* = 3 per group). (C) TBR determination of NIR-II fluorescence signal intensity in mice from (A) (*n* = 3). (D) Quantification of fluorescent signals in major organs from (B) (*n* = 3). (E and F) Representative tumor imaging in E8-IR800CW-treated HCT116 tumors before tumor excision under the NIR-II vivo imaging system. (G) Partial tumor excision under NIR-II imaging. The white arrow indicates the remaining tumor tissues underlying the removed half of tumor; the yellow arrow indicates the uncut another half of tumor. (H) Complete precise tumor removal (R0 resection) under NIR-II imaging direction. (I) Whole-body imaging of HT115 tumor-bearing mice at different time points (4, 8, 12, and 24 h) after intravenous injection with E8-IR800CW or Con-IR800CW (100 μg per mouse). (J) Ex vivo imaging for major organs dissected from in vivo-imaged mice from (I) at 24 h (*n* = 3 per group). (K) TBR of NIR-II fluorescence signal intensity in mice from (I) (*n* = 3). (L) Quantification of fluorescent intensity in major organs from (J) (*n* = 3). (M and N) Representative tumor imaging in E8-IR800CW-treated HT115 tumors before surgery. (O) Partial tumor excision under NIR-II imaging in an E8-IR800CW-treated HT115 tumor. (P) Complete tumor excision (R0 resection) directed under NIR-II imaging. *P < 0.05, **P < 0.01, and ***P < 0.001.

After successfully conjugating E8 Nb with IR800CW and confirming the binding specificity and activity of E8-IR800CW to CDH17 in CRC cells, we subsequently assessed the in vivo performance of E8-IR800CW for CRC tumor imaging and imaging-guided precise tumor removal under NIR-II window. First, a subcutaneous CRC tumor model was induced with CDH17-expressing CRC cell line HCT116. Tumor-bearing mice were intravenously administrated with 100 μg of E8-IR800CW or Con-IR800CW when the tumors reached approximately 500 mm^3^. NIR-II fluorescence images of the whole body were acquired by the MARS system to assess the tumor-specific accumulation and the in vivo distribution of the fluorescence probes. The fluorescence signal was recorded at 4, 8, 12, and 24 h respectively after probe administration (Fig. 3A). Due to the unspecific distribution of probes in the first 4 h, there were no difference between mice receiving E8-IR800CW or Con-IR800CW. The liver and kidneys appeared to show strong fluorescent signals for both groups (Fig. 3A and C). However, tumors from mice treated with E8-IR800CW indicated the gradually increased signals 8 h after probe injection compared with tumors treated with Con-IR800CW, which did not show an obvious increase in signals over time. The fluorescence intensity of tumors treated with E8-IR800CW reached a peak at 8 h and was decreased slightly at 24 h. However, the tumor-to-background ratio (TBR) for E8-IR800CW-treated tumors was gradually elevated during 24 h after probe administration due to a decrease in signal from the background organs such as skin, livers, and kidneys. After 24 h, the animals were euthanized and various organs including heart, liver, spleen, lung, kidney, and tumor were collected for ex vivo imaging under MARS. As shown in Fig. 3B and D, the strongest fluorescent signals were detected in the livers and kidneys in both groups, indicating that Nb-IR800CW probes were mainly metabolized in these 2 organs. Tumors receiving E8-IR800CW treatment showed significantly elevated signals as compared to tumors given Con-IR800CW probes, suggesting the strong targeted ability of E8 Nb against CDH17-expressing CRC tumors. Further immunostaining with VHH plus hemagglutinin (HA) antibodies for Nb detection in tumors and control organs disclosed that Nbs were detectable in tumors treated with E8-IR800CW probes, and no detectable signals were found in control organs except livers and kidneys (Fig. S4). Likewise, the Nb signal was hardly identified in the control tumors receiving the treatment with Con-IR800CW, which aligned with the weak imaging signal produced in those control tumors after Con-IR800CW injection (Fig. S4). Given the best TBR value at 24 h for E8-IR800CW-treated tumors despite the slight reduction in fluorescent signals, we next conducted the NIR-II fluorescent imaging-guided precise tumor resection in E8-IR800CW-treated tumor-bearing mice. As indicated in Fig. 3E and F, the subcutaneous tumors could be clearly identified 24 h after the injection of the E8-IR800CW probe, and livers also showed bright signals, indicating the excellent tissue penetration depth of NIR-II signals. Next, the half of tumor was first excised under the NIR-II system and removed tumor mass showed bright signals (Fig. 3G). Due to the incomplete tumor resection, the remaining tumor tissues under the excised half tumor could also be clearly observed as indicated with the white arrow (Fig. 3G); the uncut another half of tumor retained the bright signals indicated by the yellow arrow (Fig. 3G). After the complete excision of the tumor tissues, no tissues with obvious fluorescent signals were left in the skin (Fig. 3H), suggesting that E8-IR800CW could effectively direct the precise tumor removal under the NIR-II system and may achieve R0 resection to a great extent. To make the findings more reliable and generable for CDH17-expressing CRC, another CRC xenograft model initiated by HT115 was further exploited to confirm the imaging capability and fluorescence-guided surgery for E8-IR800CW. Similar results were obtained in this model as well, showing that E8-IR800CW exhibited excellent tumor imaging performance with the best TBR in tumors 24 h after probe injection (Fig. 3I and K). Ex vivo imaging for excised organs unraveled the results resembling HCT116 tumors, and tumors receiving targeted probes displayed enhanced fluorescent signals relative to control tumors treated with Con-IR800CW. The strong unspecific signals were also found in livers and kidneys (Fig. 3J and K). For imaging-guided surgery (Fig. 3M to P), E8-IR800CW could exert the prominent precise tumor excision directed by NIR-II bright signals in HT115 tumors and fulfill the R0 resection as illustrated in HT115 tumors. To assess the biosafety of the probes, we performed hematoxylin and eosin staining for major organs in HCT116 tumor-bearing mice after tumor imaging. Histological analysis did not reveal any significant morphological changes in E8-IR800-treated mice when compared with untreated mice (Fig. S5). Taken together, these results for NIR-II imaging and fluorescence-guided precise tumor excision suggest that E8-IR800CW can specifically image CRC tumors expressing CDH17 and can potentially be used to guide surgical resection.

### The E8-PE38 immunotoxin efficiently inhibits CRC in vitro

**Fig. 4.**
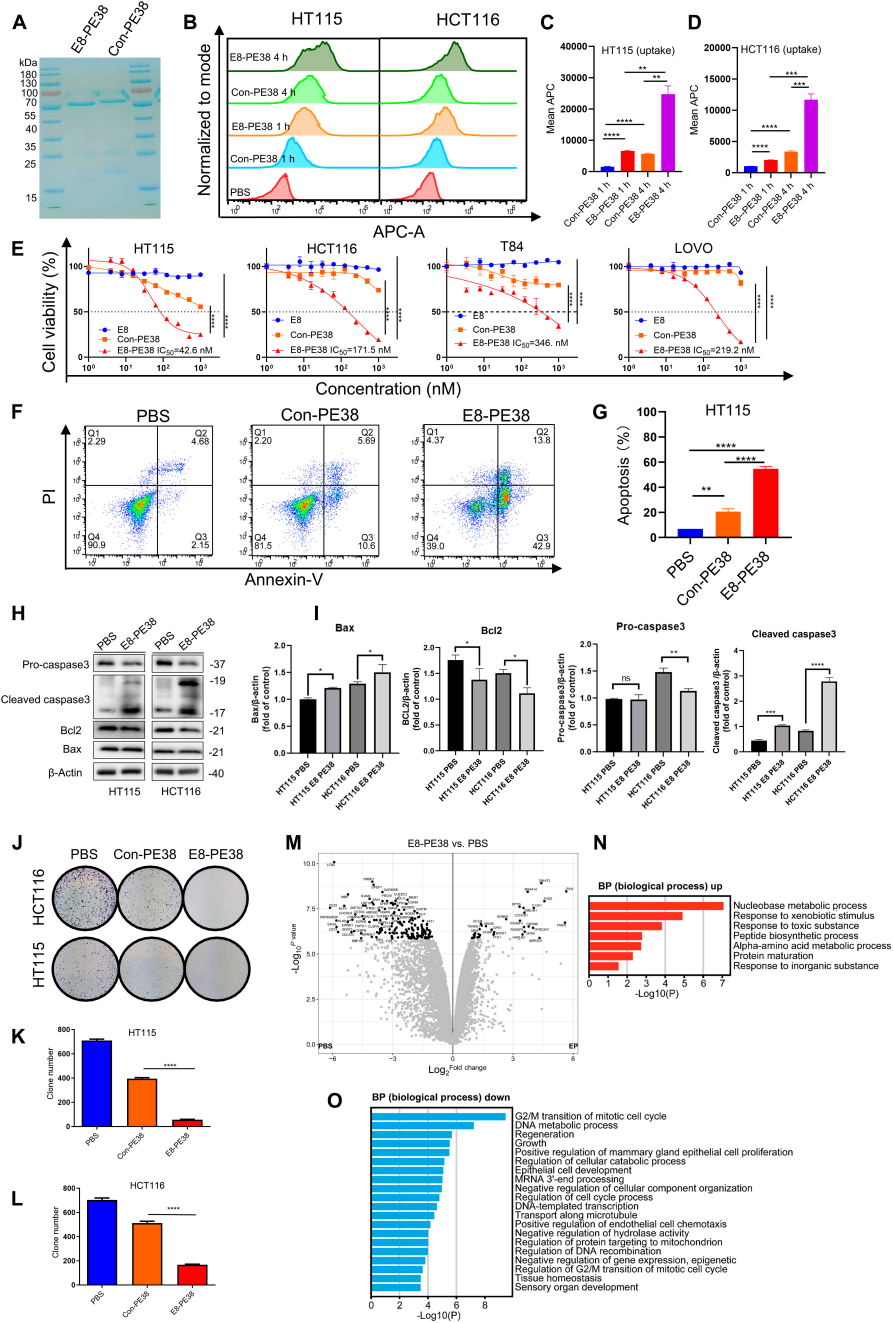
The immunotoxin E8-PE38 effectively inhibits CRC cell proliferation in vitro. (A) SDS-PAGE analysis of purified E8-PE38 and Con-PE38. (B) Flow cytometry detection of E8-PE38 and Con-PE38 internalization at different time points (1 and 4 h) in CRC cell lines (HCT116 and HT115). (C and D) Quantification of cellular uptake of E8-PE38 and Con-PE38 for (B) (*n* = 3). (E) Cell viability assay of CRC cell lines (HT115, HCT116, T84, and LOVO) treated with E8, E8-PE38, and Con-PE38 (*n* = 3). (F) Apoptotic analysis with flow cytometry in HT115 cells treated with PBS, E8-PE38, and Con-PE38. (G) Quantification of apoptotic HT115 cells for (F) (*n* = 3). (H) Western blot analysis for apoptotic protein markers in HT115 and HCT116 cells treated with PBS and E8-PE38. (I) Quantification of Western blot data for (H) (*n* = 3). (J) Colony formation analysis to determine the proliferation in HT115 and HCT116 cells treated with PBS, Con-PE38, and E8-PE38. (K and L) Quantification of colony formation in HT115 and HCT116 cells treated with PBS, Con-PE38, and E8-PE38. (*n* = 3). (M) Volcano plot of protein mass spectrometry analysis in HT115 cells treated with PBS and E8-PE38(*n* = 3). (N) Up-regulated BPs in HT115 cells after treatment with E8-PE38 immunotoxin. (O) Down-regulated BPs in HT115 cells after treatment with E8-PE38 immunotoxin. *P < 0.05, **P < 0.01, and ***P < 0.001.

Considering that the E8-IR800CW NIR-II fluorescent probe exhibits outstanding tumor imaging capability and effectively directs imaging-guided precise tumor excision in CDH17-overexpressing CRC models, we next investigated whether CDH17-targeting Nb E8 fused with toxin PE38 could inhibit CRC. The immunotoxin E8-PE38 and Con-PE38 were purified as described previously [13], and the soluble recombinant immunotoxins were obtained with high purity (Fig. 4A). The binding activity of E8-PE38 and Con-PE38 proteins to CDH17 was detected by ELISA, showing that PE38 did not alter the binding ability of E8 Nb against CDH17 protein, and Con-PE38 as a negative control could not interact with CDH17 (Fig. S6). The binding affinity (*K*_D_) of E8-PE38 to CDH17 has been determined to be 86.87 nM comparable to the affinity of E8 to CDH17 (70.3 nM) [13]. Given that the efficient internalization is essential for immunotoxin to exert an inhibitory effect on cancer cells, and E8 Nb could be rapidly internalized by CRC cells (Fig. 2D), flow cytometry analysis with Cy5-labeled E8-PE38 or Con-PE38 uncovered that E8-PE38 immunotoxin could be efficiently ingested in both CRC cells HT115 and HCT116 when compared with Con-PE38, and internalization of E8-PE38 was significantly increased over time, whereas untargeted Con-PE38 indicated the internalization with low efficiency (Fig. 4B to D). Toxin PE38 could effectively suppress cancer cell viability through the inhibition of protein synthesis once internalized [27]. We subsequently assess the cell viability after treatment with E8 Nb, E8-PE38, and Con-PE38 in 4 CRC cells HT115, HCT116, LOVO, and T84 (Fig. 4E). As shown in Fig. 4E, E8-PE38 immunotoxin effectively repressed the cell viability in all the 4 CRC cells tested with various IC_50_ from 42.6 to 346 nM, whereas Con-PE38 just slightly inhibited the cell viability due to the unspecific uptake, and E8 alone did not show any inhibitory effect on cell viability. These data showed that E8 Nb could deliver toxin PE38 to CDH17-positive CRC cells and significantly enhance the internalization of PE38, which in turn kills the CRC cells. Further apoptosis analysis with flow cytometry demonstrated that E8-PE38 could markedly increase apoptotic cell number compared with Con-PE38 or vehicle control in both cell lines (Fig. 4F and G and Fig. S7). The percentage of apoptosis (annexin V^+^ cells plus annexin V^+^/PI^+^ cells) in the E8-PE38-treated HT115 and HCT116 were 54.77 ± 1.785% and 37.20 ± 0.251%, respectively, which were much higher than those in the Con-PE38-treated corresponding control cells (20.49 ± 2.331% and 15.84 ± 0.234%) (Fig. 4F and G and Fig. S7). The analysis of apoptotic marker proteins with Western blotting showed that the relative expression levels of pro-apoptotic protein Bax and cleaved caspase-3 were significantly increased, and antiapoptotic protein Bcl2 was obviously decreased in E8-PE38-treated HT115 or HCT116 cells, indicating that E8-PE38 immunotoxin can induce the apoptosis of CRC cells in vitro (Fig. 4H and I). Cell colony formation assay also indicated that E8-PE38 significantly inhibited cell proliferation, while Con-PE38 had little effect in both CRC cell lines (Fig. 4J to L). To globally decipher the impact of immunotoxins on protein synthesis, by which cell death is initiated, proteomics analysis in HT115 cells treated with E8-PE38 was performed to disclose the global alterations in proteins. The data showed significant changes in a total of 204 proteins (Fig. 4M and Table S1). Among the proteins identified, 37 proteins were up-regulated, while 167 proteins were down-regulated after treatment in HT115 with E8-PE38, as illustrated in Fig. 4M. Subsequently, biological process (BP) analysis revealed the relative pathway enrichments for the corresponding up- and down-regulated proteins. E8-PE38 generally enhanced the responses of cells against xenobiotic stimulus, toxic substances, and inorganic substances (Fig. 4N); accordingly, E8-PE38 immunotoxin down-regulated a multiple of BPs such as cell cycle, DNA metabolic process and transcription, regeneration, and cell proliferation (Fig. 4O). It seemed that E8-PE38 could produce a complicated influence on intracellular signal networks upon the initiation of protein synthesis inhibition by PE38 toxin. Collectively, these results about E8-PE38 immunotoxin in CRC cells indicate that CDH17 Nb E8 could enhance the inhibitory effect of PE38 on CRC cells through CDH17-mediated internalization, and E8-PE38 immunotoxin can induce CRC cell apoptosis through the complicated influence on intracellular signal networks, suggesting the great potential for E8-PE38 immunotoxin in the treatment of CRC in vivo.

### E8-PE38Mut immunotoxin imaging shows outstanding tumor accumulation in vivo

**Fig. 5.**
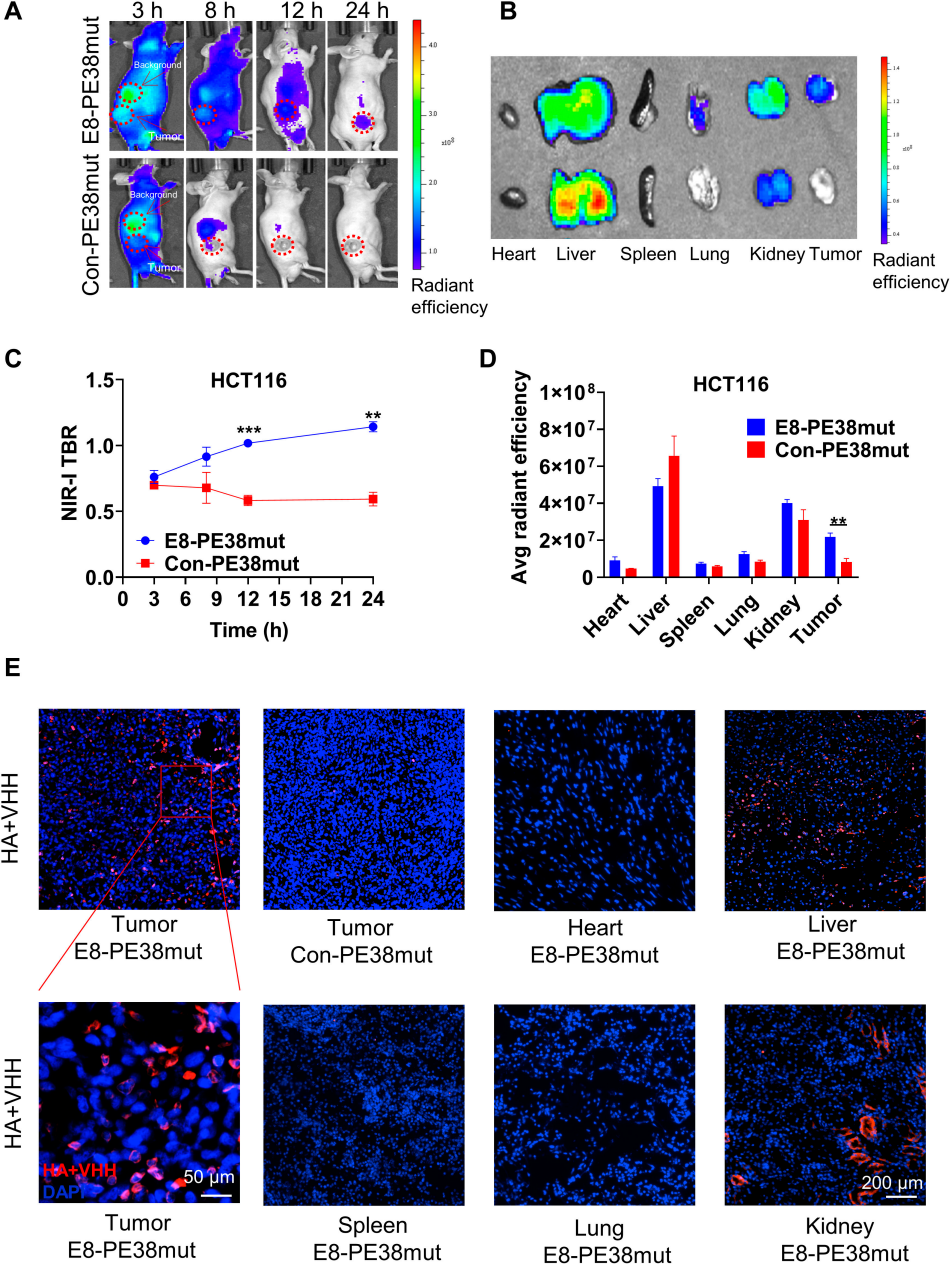
In vivo imaging examination for inactive immunotoxins. (A) Imaging of HCT116 tumors in mice at different time points (3, 8, 12, and 24 h) after intravenous injection with E8-PE38mut-IR800CW and Con-PE38mut-IR800CW (200 μg per mouse). (B) Ex vivo imaging of major organs dissected from in vivo-imaged mice from (A) at 24 h (*n* = 3 per group). (C) Quantification of TBR in mice based on NIR-I fluorescence signal intensity from (A) (*n* = 3). (D) Quantification analysis of inactive immunotoxin distribution in major control organs and tumors from (B) (*n* = 3). (E) Immunofluorescence staining for various tissues collected at 24 h after intravenous injection to determine immunotoxin distribution. Scale bar, 200 or 50 μm. E8-PE38mut-IR800CW specifically accumulated in CDH17-positive tumor tissues. Liver and kidney tissues showed weak staining due to nonspecific phagocytosis and metabolism. *P < 0.05, **P < 0.01, and ***P < 0.001.

Although PE-based immunotoxins have been extensively investigated in various preclinical and clinical settings, and moxetumomab pasudotox, a PE-based immunotoxin targeting CD22, has been approved by the US Food and Drug Administration (FDA) to treat hairy cell leukemia [28], few studies have clearly demonstrated through tumor imaging that immunotoxins could be specifically delivered to tumor tissues, which is crucial for immunotoxin to exert the antitumor functions. To evaluate the specific accumulation of E8-PE38 immunotoxin in CRC tumors, inactive immunotoxins E8-PE38Mut and Con-PE38Mut were first constructed through the introduction of the mutation E553D [29] in PE38 toxin molecule and further purified because a relatively large amount of immunotoxins (200 μg per mouse) are needed for imaging detection and the active immunotoxins under this dosage are lethal to tumor-bearing mice [29]. The soluble mutant immunotoxin proteins were purified from *Escherichia coli* and showed a molecular weight of approximately 60 kDa as expected (Fig. S8A). The purified mutant immunotoxins were then labeled with IR800CW, and the IR800CW-labeled mutant immunotoxins retained the same binding activity as the corresponding unlabeled proteins, indicating that IR800CW labeling did not change the binding property of E8-PE38Mut against CDH17 protein (Fig. S8B). Subsequently, E8-PE38Mut-IR800CW and Con-PE38Mut-IR800CW (200 μg per mouse) were intravenously injected into HCT116 tumor-bearing mice and the whole-body images were captured with NIR-I IVIS Imaging system under 4 time points. As shown in Fig. 5A, E8-PE38Mut-IR800CW gradually accumulated into tumors over time after administration. It showed significant higher TBR signals at 12 h than Con-PE38Mut-IR800CW and retained the high TBR until 24 h, whereas nontargeted Con-PE38Mut-IR800CW did not exhibit any specific accumulation in tumors besides liver areas and signals almost disappeared 24 h after drug administration, manifesting that CDH17-targeting Nb E8 could deliver the PE38 toxin into CRC tumors specifically and efficiently (Fig. 5A and C). After imaging, ex vivo fluorescence imaging for major control organs and tumors was conducted to further explore the biodistribution of the immunotoxins. E8-PE38Mut-IR800CW exhibited higher fluorescence signals in the tumor tissues, livers, and kidneys; however, high fluorescence signals for Con-PE38Mut-IR800CW were mainly detected in the livers and kidneys (Fig. 5B and D). These data were consistent with the whole-body imaging, further implying the E8-PE38mut specificity to CDH17-positive CRC tumors. Immunofluorescence staining with VHH plus HA antibodies to detect the Nbs in various tissues could detected strong positive signals in tumor tissues treated with E8-PE38mut, and weak staining could also be identified in livers and kidneys due to the phagocytosis and excretion to immunotoxins, while no signal was found in tumor tissues treated with Con-PE38mut, suggesting that E8-PE38Mut was highly enriched in tumors, which is consistent with the data from in vivo and ex vivo imaging (Fig. 5E). Together, the immunotoxin imaging results here demonstrate that CDH17 Nb E8 can specifically deliver inactive toxin PE38mut in tumors positive for CDH17 expression, implicating that, therefore, active immunotoxin, E8-PE38, can efficiently home to CDH17-positive CRC tumors and execute in vivo antitumor functions.

### Active E8-PE38 immunotoxin significantly inhibits CRC growth in vivo

**Fig. 6.**
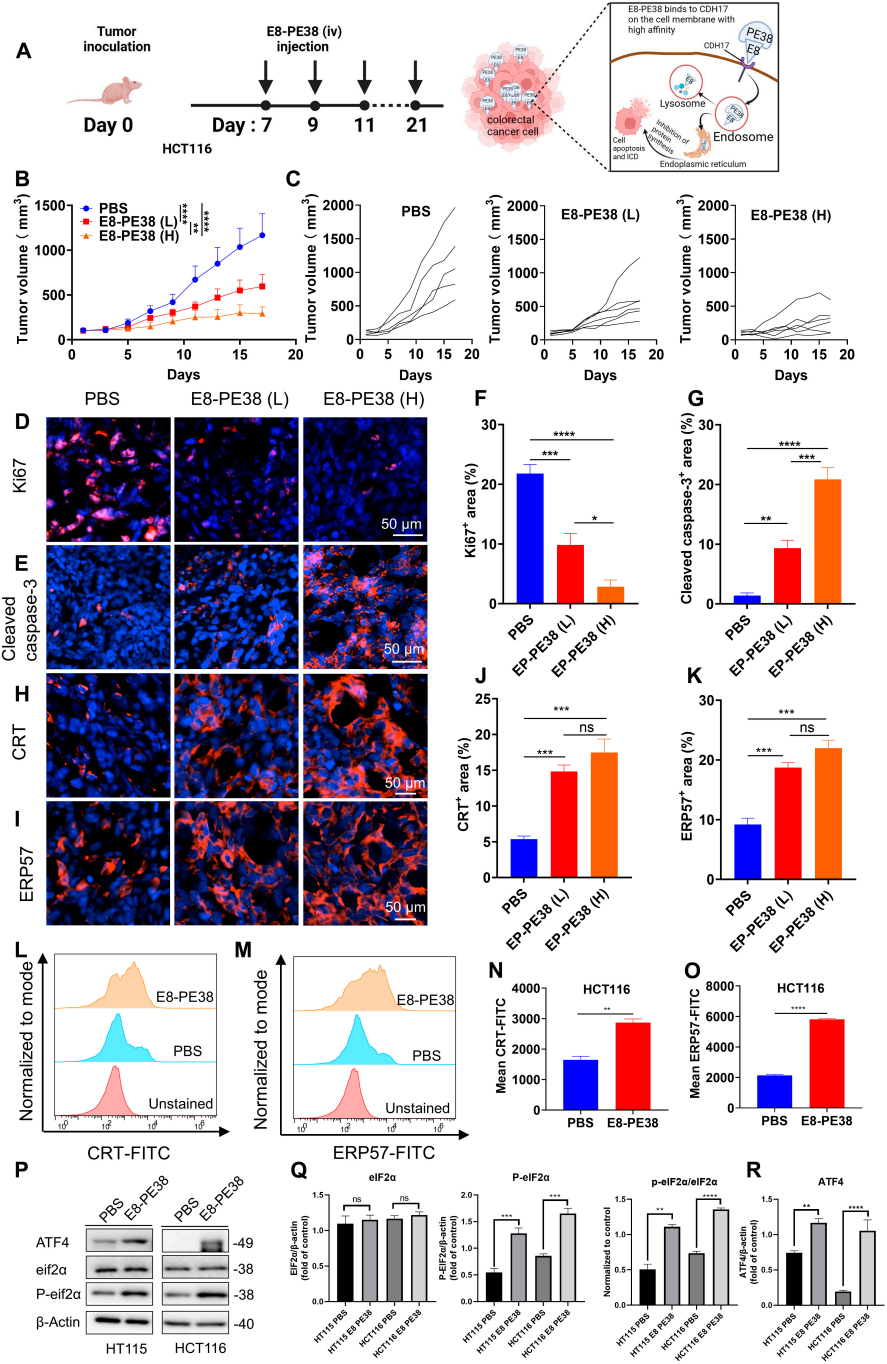
Evaluation of the antitumor effects of the E8-PE38 immunotoxin in vivo. (A) Schematic therapeutic schedule and mechanisms of E8-PE38 (0.6 and 0.8 mg/kg) for the treatment of HCT116 subcutaneous tumors. (B) Tumor growth curves of HCT116 tumors after treatment with PBS and 0.6 or 0.8 mg/kg of E8-PE38 (*n* = 5 to 6 per group). (C) Individual tumor growth curves from 3 groups in (B). (D) Ki67 immunofluorescence staining in HCT116 tumors after immunotoxin treatment. Scale bar, 50 μm. (E) Cleaved caspase-3 immunofluorescence staining in HCT116 tumors from 3 groups. Scale bar, 50 μm. (F) Quantitative analysis of Ki67 expression for (D) (*n* > 3). (G) Quantitative analysis of cleaved caspase-3 expression for (E). (H) CRT immunofluorescence staining in tumors from 3 groups treated with PBS or immunotoxin. Scale bar, 50 μm. (I) ERp57 immunofluorescence staining in tumors from 3 groups treated with PBS or immunotoxin. Scale bar, 50 μm. (J) Quantitative analysis of CRT expression for (H) (*n* = 3). (K) Quantitative analysis of ERp57 expression for (I) (*n* = 3). (L) Detection of CRT expression on cell membrane with flow cytometry after E8-PE38 treatment in HCT116 cells. (M) Detection of ERp57 expression on cell membrane with flow cytometry after E8-PE38 treatment in HCT116 cells. (N) Quantitation for CRT expression in (L) (*n* = 3). (O) Quantitation for ERp57 expression in (M) (*n* = 3). (P) Western blot analysis for ATF4, eIF2α, and p-eIF2α expression in HT115 and HCT116 cells treated with PBS and E8-PE38. (Q) Quantification for ATF4, eIF2α, and p-eIF2α expression in HT115 and HCT116 cells treated with PBS and E8-PE38 in (P) (*n* = 3). (R) Quantification for ATF4 expression in HT115 and HCT116 cells treated with PBS and E8-PE38 in (P) (*n* = 3). *P < 0.05, **P < 0.01, ***P < 0.001.

Given the excellent in vitro cytotoxic effect of E8-PE38 and specific accumulation of inactive E8-PE38Mut into CDH17-positve CRC tumors, the actual antitumor performance for active immunotoxin E8-PE38 was then explored in an HCT116-induced subcutaneous model. Due to the high toxicity of nontargeted PE38 to tumor-bearing mice and no obvious tumor inhibition for E8/E8-PE38mut in vitro or in vivo, these 3 proteins were not included in this study [13]. Two doses of E8-PE38 (0.6 and 0.8 mg/kg) were evaluated in the HCT116 tumor model when tumors were approximately 100 mm^3^. The therapeutic schedule was shown in Fig. 6A. Both doses significantly suppressed tumor growth when compared with the vehicle control (Fig. 6B). The higher dosage displayed a more significant tumor inhibition effect and resulted in almost complete cessation of tumor growth, suggesting that E8-PE38 has a very strong inhibitory effect on tumor growth (Fig. 6C). Meanwhile, there was no significant change in body weight during the whole therapeutic period, indicating good biosafety for E8-PE38 immunotoxin (Fig. S9). To ascertain the inhibitory impact of E8-PE38 on HCT116 tumors, the additional evaluation for cell proliferation and apoptosis via Ki67 and cleaved caspase-3 immunohistochemical staining was carried out (Fig. 6D and E). The results showed that Ki67 expression in tumor tissues was significantly reduced after treatment with immunotoxin E8-PE38 as compared to that in the PBS group (Fig. 6F). Furthermore, the cleaved caspase-3 staining confirmed that the immunotoxin induced apoptosis in a considerable number of cancer cells, while no significant apoptosis was observed in the control group (Fig. 6G). These findings suggest that the immunotoxin E8-PE8 can prevent CRC tumor growth by inhibiting cell proliferation and promoting apoptosis. Subsequent systemic toxicological assessments were conducted to ensure the biosafety of the immunotoxin E8-PE38 after treatment. Hematology and serum biochemistry analysis revealed no significant variations in mice treated with E8-PE38 compared to the PBS group, except for slight elevation in alanine aminotransferase and white blood cells (Fig. S10A and B), which was consistent with previous reports [29,30]. Histological analysis of major organs did not reveal any significant morphological changes in any of the groups (Fig. S11).

In addition to apoptosis induced by immunotoxins, the ability of immunotoxins to induce ICD has already been reported and found even in cancer patients [31], and ICD in tumor cells can activate the systemic antitumor immune response through the interaction of damage-associated molecular patterns (DAMPs) with immune cells such as dendritic cells and macrophages. The translocation of DAMP calreticulin (CRT) and ERp57 from the endoplasmic reticulum (ER) to the cell surface is an early marker of ICD in tumor cells, with both proteins playing a pivotal role in activating antitumor immunity. Therefore, we further evaluated whether immunotoxin E8-PE38 could induce ICD in CRC tumor cells both in vivo and in vitro. First, the membrane expression of CRT and ERp57 in HCT116 tumor tissues receiving E8-PE38 treatment was up-regulated more than that of tumors in the PBS group (Fig. 6H to K). In vitro cell flow cytometry analysis after treatment with E8-PE38 confirmed the induction of ICD, showing a significant increase for the expression of CRT and ERp57 in treated HCT116 and HT115 cells (Fig. 6L to O and Fig. S12A and B). ICD induction is highly associated with ER stress, and PE38-based immunotoxin could be retained in ER through KDEL motif and intervene with protein synthesis [32]. Hence, we further examined the changes of ER stress-related proteins after E8-PE38 treatment in HT115 and HCT116 cells. E8-PE38 treatment could up-regulate the cellular ATF4 and phosphorylated eIF2α protein, both of which are indicators for ER stress (Fig. 6P to R). Taken together, these results demonstrate that the E8-PE38 immunotoxin can efficaciously inhibit CRC tumor development in vivo by promoting apoptosis and inducing ICD in tumor cells.

### E8-PE38 immunotoxin synergistically inhibits CRC tumors with the standard chemo-drug 5-FU

**Fig. 7.**
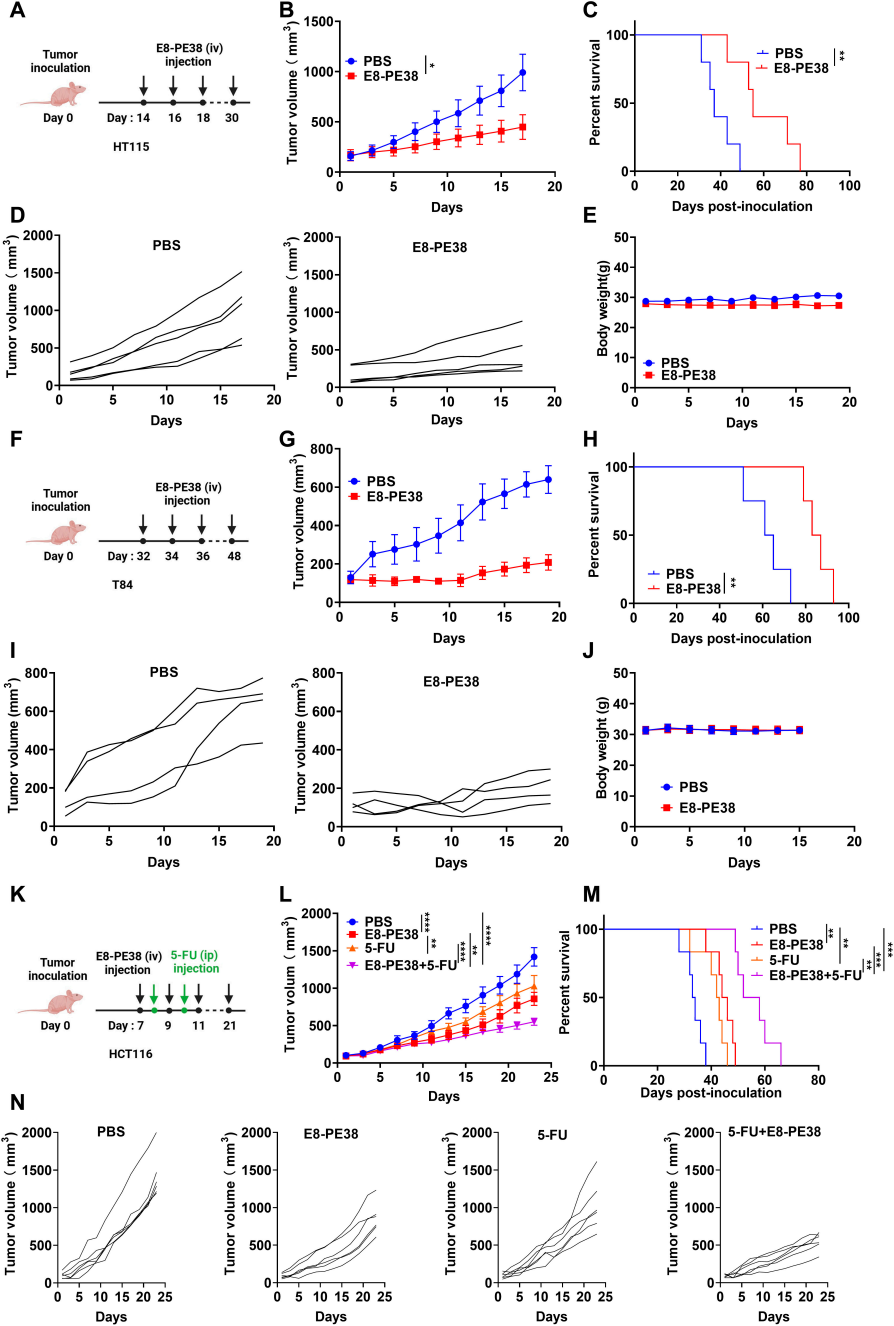
The antitumor effects of the immunotoxin E8-PE38 combined with 5-FU. (A) Therapeutic schedule of E8-PE38 (0.4 mg/kg) as a monotherapy for the subcutaneous HT115 tumors. (B) HT115 tumor growth curves in the mice receiving treatment with PBS and 0.4 mg/kg of E8-PE38 (*n* = 5 per group). (C) Tumor survival analysis for mice in (B). (D) Individual tumor growth curves from HT115 tumor-bearing mice in (B). (E) Body weight of the mice during the treatment in (B). (F) Therapeutic schedule diagram of E8-PE38 (0.6 mg/kg) for T84 subcutaneous tumors. (G) T84 tumor growth curves with treatment with PBS and 0.6 mg/kg of E8-PE38 (*n* = 4 per group). (H) Tumor survival analysis for mice in (G). (I) Individual tumor growth curves from T84 tumor-bearing mice in (G). (J) Body weight for the mice during treatment in (G). (K) Combination therapeutic schedule for E8-PE38 (0.6 mg/kg) and 5-FU (25 mg/kg) in HCT116 subcutaneous tumor model. (L) HCT116 tumor growth curves after treatment with PBS, 25 mg/kg 5-FU, 0.6 mg/kg E8-PE38, and the combination (*n* = 6). (M) Tumor survival analysis for mice in (L) (*n* = 6 per group). (N) Individual tumor growth curves from HCT116 tumor-bearing mice receiving various treatments in (L). *P < 0.05, **P < 0.01, and ***P < 0.001.

To further extend the application of E8-PE38 immunotoxin in CDH17-positive CRC and confirm the prominent tumor suppression activity by E8-PE38, another 2 CRC models induced by HT115 and T84 cells were employed to test the antitumor effect of E8-P38 immunotoxin. For the HT115 model, a dose of 0.4 mg/kg E8-PE38 significantly inhibited the tumor growth and prolonged the survival time (Fig. 7A to D). There was no significant change in body weight in the whole treatment period (Fig. 7E). Similarly, a dose of 0.6 mg/kg E8-PE38 showed superb antitumor efficacy in T84-induced tumor model and remarkably extended the mice survival without obvious change in terms of body weight (Fig. 7F to J). The potent antitumor activity of E8-PE38 in these 2 CRC models further demonstrates that CDH17 Nb-targeted immunotoxin holds great potential for CRC treatment.

Clinically, a 5-FU-based chemotherapeutic regimen is used as a first-line strategy to treat CRC patients with certain characteristics such as intact RAS and BRAF [33,34]. The primary mechanism of action of 5-FU is to inhibit DNA synthesis, ultimately leading to cell death [35,36]. The combination therapy is normally a common option for clinical practice or new drug trials. Therefore, we finally determined whether combined immunotoxin with 5-FU could produce synergistic tumor suppression in the HCT116 subcutaneous tumor model (Fig. 7K). High dose of 5-FU (50 mg/kg) by daily intraperitoneal injection in mice could cause intestinal injury, but a low dose of 5-FU (25 mg/kg ) did not show any side effects including loss of body weight, diarrhea, and intestinal injury [37,38]. Therefore, a low dosage for both drugs was selected to maximize the combinational efficacy and minimize the potential side effects. Low doses of 5-FU (25 mg/kg) and E8-PE38 (0.6 mg/kg) alone showed fairly similar antitumor effects, although it seemed that 0.6 mg/kg E8-PE38 alone worked a bit better than 25 mg/kg 5-FU, whereas the combination treatment exhibited maximal tumor growth inhibition among all the groups and survival extension (Fig. 7L to N). No significant change in body weight was detected across all the treatments (Fig. S13), indicating that the combination therapy under low dosages is tolerable to animals with good biological safety.

Taking all the therapeutic data together, we demonstrate that E8-PE38 immunotoxin shows great potential for CRC treatment, and immunotoxins targeting CDH17 might be developed as a novel adjuvant agent combined with standard drugs to treat CDH17-positive CRC.

## Discussion

The preoperative assessment and intraoperative visualization are critical to design surgery plans and precisely resect tumors for oncological surgery. Fluorescence-guided surgical tumor resection has emerged as a valuable tool for surgeons, enabling the differentiation of tumor margins from normal tissues and identification of lymph nodes with metastatic tumor cells during operation. This approach facilitates precise tumor excision while preserving healthy tissues. Since indocyanine green (ICG) was approved for clinical application for intraoperative imaging [6], fluorescence-guided surgery navigation has been largely developed and extensively applied in clinical practice. However, ICG with high biological safety lacks tumor selectivity and has difficulty in visualizing deep tissues within NIR-I (400 to 900 nm) window [8]. Therefore, it is warranted to develop tumor-specific fluorescent probes and advanced imaging systems with deeper tissue penetration and higher spatial resolution for surgery navigation. Compared to NIR-I imaging, NIR-II (1,000 to 1,700 nm) imaging provides more advantageous properties for clinical practice, such as low autofluorescence, deeper tissue penetration, and high imaging contrast and resolution, making NIR-II imaging more attractive in preclinical and clinical settings in recent years [5,6,10]. Moreover, due to the findings that ICG or IR800CW normally used as NIR-I fluorescent molecules could show long emission tails in NIR-II window, the rapid clinical translation could be achieved based on the wide applications having proved their efficacy and biosafety. Conventional monoclonal antibodies have been exploited to conjugate with fluorescence imaging molecules to obtain tumor selectivity, and realize precise tumor imaging and resection, such as SGM101, a CEA monoclonal antibody conjugated with fluorochrome BM104 for fluorescence-guided surgery to detect CRC and metastasis [39]. However, full-length monoclonal antibodies with 150 kDa could retain high concentration in the circulation, resulting in a relatively long half-life, low accumulation, and delayed fluorescent peak time in tumor sites, which are unfavorable for use in clinical practical [7,40]. In contrast, Nbs, which are the smallest antibody entities, have been recently given more attention to develop fluorescent probes for surgery navigation owing to their small molecular weight, good tissue penetration, easy preparation with high solubility, and moderate half-life relative to full-length antibodies or tiny peptides. In comparison to monoclonal antibody-based fluorescent probes, Nb-modified imaging agents could realize rapid imaging and same-day examination after administration, making them more favorable and suitable for clinical settings [41,42]. Notably, targeted CEA Nb conjugated with IR800CW has been assessed for NIR-II CRC imaging and surgery navigation in the preclinical settings, showing the advantages of Nb-based probes [7]. However, to our knowledge, no studies have explored the use of NIR-II probes with Nbs targeting CDH17 for CRC imaging and imaging-guided surgery. In this study, we utilized a CDH17-targeting Nb E8, identified by our group, conjugated with fluorescence molecule IR800CW with a NIR-II tail to examine its performance for CRC imaging and precise tumor removal. Our results demonstrate that E8-IR800CW can efficaciously detect the CRC tumor mass and guide the precise tumor excision. The probe showed a high TBR starting from 12 h after injection and remained detectable for up to 24 h, providing a suitable time window for surgery conduction. In addition, the peak time for NIR-II fluorescent signals occurs around 12 h after injection, making it more practical and convenient for patients compared to monoclonal antibody-based probes, which normally require administration 3 to 4 days prior to surgery [39]. Primary CRC commonly metastasizes to liver, lung, and peritoneal cavity, and it is still challenging to visualize metastatic CRC tissues and distinguish them from nearby healthy tissues. Given the maintenance of high expression of CDH17 in metastatic CRC [14], future studies should investigate the potential of CDH17 Nb-based NIR-II probes for imaging metastatic CRC tissues and direct the imaging-guided metastatic tumor removal. In addition, since both ICG and IR800CW were not originally developed as NIR-II imaging probes. They just exhibit a NIR-II region tail and, thus, can be applied for NIR-II imaging. It is warranted to conjugate E8 Nb with other more specific NIR-II probes such as Flav7, IR-1061, or conjugated polymer nanoparticles (CP NPs) with NIR-II property [5,9], which might result in deeper tissue penetration and better tumor imaging quality, even for those tumors located in peritoneal cavity such as orthotopic CRC tumors. Moreover, the development of multimodal imaging system with Nbs conjugated with novel multifunctional probes such as CP NPs with NIR-II and photoacoustic properties is another important strategy to realize precise tumor diagnosis or accomplish theragnostic applications [10].

Although great improvement has been obtained for CRC drug therapy besides surgery in the past decades, especially the applications of checkpoint blockades (antibodies against PD-1 and PD-L1) in CRC therapeutic regimens in addition to 5-FU-based chemo-drugs, EGFR and VEGF antibodies, and small molecules targeting mutant KRAS and BRAF [43,44], the prognosis for patients with advanced and metastatic CRC remains suboptimal. Immunotoxins as a type of antibody-conjugated drugs consist of TAAs targeting units such as antibodies, antibody fragments, Nbs, or peptides fused with various truncated toxins without tumor selection [27]. A diversity of immunotoxins has been broadly explored in various tumors including CRC preclinically and clinically, such as FDA-approved moxetumomab pasudotox and ongoing clinical trial RG7787, both of which are PE-based immunotoxins targeting CD22 and mesothelin, respectively [27,28]. However, most studies focus on the antitumor performance without clearly demonstrating efficient delivery of toxin proteins to tumor tissues with visible imaging strategies. In this study, we directly labeled the toxin-inactivated E8-PE38Mut with fluorescence dye and performed tumor imaging. Indeed, E8-PE38Mut showed specific accumulation in the CRC tumors, indicating that tumor targeting unit E8 Nb could efficiently carry the toxin to tumor sites and exert antitumor activity. To the best of our knowledge, this is the first report to show that a PE-based immunotoxin is specifically imaged in tumor tissues mediated by Nb targeting against CDH17 in CRC. This finding is of great significance for immunotoxin assessment as anticancer agents and highlights the importance of imaging for ensuring the specific tumor selection and targeting for immunotoxins.

Apoptosis induced by PE-based immunotoxins through the inhibition of protein synthesis is a common mechanism accepted in the past decades [29]. However, clinical trial data indicated that PE-based immunotoxins could provoke systemic antitumor immunity, and later on, PE-based immunotoxins was proved to be able to induce ICD, promote the formation of tertiary lymphoid structure, and enhance T cell infiltration and antitumor immunity [28,32]. Interestingly, a clinical trial for PE-based immunotoxin MOC31PE targeting EpCAM suggested that MOC31PE as a monotherapy could significantly extend the survival in metastatic CRC patient compared to MOC31PE plus immunosuppressive agent cyclosporin to avoid the production of antitoxin antibodies in the body [23]. Mechanistic investigation disclosed that MOC31PE can potentially induce ICD and promote the production of inflammatory cytokines for immune activation. ICD has been well documented to activate adaptive immune response, and intracellular stress, such as ER stress, is highly associated with the induction of ICD [45]. These findings further imply that PE-based immunotoxins could exert immuno-activation functions and might improve the response to immunotherapy, especially in those cancers with cold tumor microenvironment (TME). In this study, we indeed identified that E8-PE38 not only triggers apoptosis but also elicits ICD in vitro and in vivo after treatment. E8-PE38 markedly induces the translocation of CRT and ERp57, both of which serve as indicators for ICD induction as DAMPs. ER stress analysis shows that E8-PE38 results in the up-regulation of ATF4 and p-eIF2α, suggesting that ICD induction with E8-PE38 might be caused by ER stress, reminiscent of the KDEL motif in PE toxin, which could interact with ER. However, it should be noted that due to the use of immunocompromised mice in our study and the weak binding affinity of E8 Nb against mouse CDH17, we were unable to further investigate the changes in the TME. Hence, it would be of great value to identify Nbs recognizing mouse CDH17 and construct the corresponding immunotoxins to assess the TME in a syngeneic mouse CRC model induced with murine CRC cells highly expressing murine CDH17 in future studies. To avoid the potential production of antitoxin antibodies in immunocompetent mouse model, alternative PE fragments such as PE24 and T20 with reduced immunogenicity [30,46] would be favorable for immunotoxin construction.

Importantly, the combination therapy of 5-FU and E8-PE38 with lower doses obtains prominent tumor suppression. The primary antitumor mechanism of 5-FU is involved in the interference with DNA replication and repair [47], which ultimately promotes apoptosis of cancer cells, while PE38 toxin mainly induces apoptosis and ICD of tumor cells through the inhibition of protein synthesis via ER stress [32]. The combination of these 2 drugs might exert synergetic cytotoxic effects on cancer cells through the inhibition of DNA replication and protein synthesis, eventually leading to broad tumor cell death and superb suppression of tumor growth. Additionally, using the combination regimen can lower the administered amounts for both of drugs, potentially reducing the risk of side effects while enhancing the overall antitumor efficacy.

Furthermore, given the high expression of CDH17 in CRC tumors and the good imaging capability of E8-IR800CW against CDH17, fluorescent endoscopy combined with E8-IR800CW could be clinically utilized to image CDH17-positive CRCs and could potentially differentiate the tumors from healthy tissues, which might facilitate the CRC removal with endoscopic surgery, which has been widely applied for colorectal polyps [48]. Meanwhile, it is also feasible and practicable to conduct image-guided therapy. E8-IR800CW can be used to diagnose CRCs, and subsequently, E8-PE38 is employed to treat the CRCs with positive imaging. E8-IR800CW can help to clarify the expression of CDH17 and select the CRCs with CDH17-positive expression for E8-PE38 treatment, which will facilitate constructing a closed loop from diagnosis to therapy. However, an important premise is that CDH17 binding sites need to be completely released from the occupancy of E8-IR800CW prior to E8-PE38 treatment; otherwise, the therapeutic efficacy of E8-PE38 might be compromised. All the potential applications aforementioned are warranted for further investigations to accelerate the translation of CDH17 Nbs.

### Conclusion

In summary, this study provides evidence that targeting CDH17 with the Nb E8 can efficiently deliver the NIR-II probe IRDye800CW to CRC tumors and visualize the tumors with high-quality imaging. Furthermore, E8-IR800CW enables the imaging-guided surgery navigation, facilitating precise excision of CRC tumors highly expressing CDH17. Meanwhile, the E8 Nb fused with PE38 toxin can efficaciously deliver toxins to CRC tumors, as evidenced by direct toxin imaging in the tumors. Of note, E8-PE38 immunotoxin significantly suppresses the development of CRC both in vitro and in vivo through apoptosis and ICD, and synergistically enhances the antitumor effect of 5-FU. These findings highlight the importance of targeting CDH17 for CRC diagnosis and therapy. Moreover, the use of CDH17 Nbs enables imaging and surgery navigation within the NIR-II window and allows for efficient targeted therapy with toxin delivery. These results open new avenues for the potential clinical translation of CDH17 targeting in CRC.

## Data Availability

The materials and data obtained or analyzed in this study are available from the authors upon reasonable request.
