# Supplementary material for: Cadherin 17 Nanobody-Mediated Near-Infrared-II Fluorescence Imaging-Guided Surgery and Immunotoxin Delivery for Colorectal Cancer
Source: Biomater Res. 2024 Jun 21;28:0041. doi: 10.34133/bmr.0041 (PMC11192146; doi:10.34133/bmr.0041)
Supplement: Supplementary 1 — Figs. S1 to S13 Table S1 Materials and Methods [file bmr.0041.f1.zip › Revised Supplementary materials.docx]

**Supplementary Materials**

**CDH17 Nanobody**-**Mediated NIR-II Fluorescence Imaging-Guided Surgery and** **Immunotoxin Delivery for** **Colorectal Cancer**

Youbin Ding^1, 2 †^, Runhua Zhou ^3,7 †^, Guangwei Shi^2,^ ^4 †^, Yuke Jiang^2 †^, Zhifen, Li^5^, Xiaolong Xu^2^, Jingbo Ma^2^, Jingnan Huang^2^, Chunjin Fu^2^, Hongchao Zhou^2^, Huifang Wang^2^, Jiexuan Li^2^, Zhiyu Dong^2^, Qinglin Yu^1^, Kexin Jiang^1^, Yehai An^3^, Yawei Liu^4^, Yilei Li^7^, Le Yu^3*^, Zhijie Li^2*^, Xiaodong Zhang^1*^, Jigang Wang^1, 2, 3, 6, 8, 9*^

*Corresponding authors:

wangjigang@u.nus.edu (Jigang Wang),

ddautumn@126.com (Xiaodong Zhang),

li.zhijie@szhospital.com (Zhijie Li),

yule0423@gmail.com (Le Yu),

^†^These authors contribute equally to this work and share the first-authorship.

^1^Department of Medical Imaging, The Third Affiliated Hospital, Southern Medical University (Academy of Orthopedics Guangdong Province), Guangzhou, 510515, P. R. China

^2^Shenzhen Clinical Research Centre for Geriatrics and Department of Geriatrics, Shenzhen People’s Hospital; First Affiliated Hospital of Southern University of Science and Technology, Second Clinical Medical College of Jinan University, Shenzhen 518020, Guangdong, P. R. China

^3^NMPA Key Laboratory for Research and Evaluation of Drug Metabolism & Guangdong Provincial Key Laboratory of New Drug Screening & Guangdong-Hongkong-Macao Joint Laboratory for New Drug Screening, School of Pharmaceutical Sciences, Southern Medical University, Guangzhou 510515, P. R China

^4^Department of Neurosurgery & Medical Research Center, Shunde Hospital, Southern Medical University (The First People's Hospital of Shunde Foshan), Guangzhou 510515, P. R. China

^5^School of Chemistry and Chemical Engineering, Shanxi Datong University, Xing Yun Street, Pingcheng District, Datong, Shanxi Province 037009, P. R. China

^6^State Key Laboratory for Quality Ensurance and Sustainable Use of Dao-di Herbs, Artemisinin Research Center, and Institute of Chinese Materia Medica, China Academy of Chinese Medical Sciences, Beijing 100700, P. R. China

^7^Department of Pharmacy, Nanfang Hospital, Southern Medical University, Guangzhou 510515, P. R China.

^8^State Key Laboratory of Antiviral Drugs, School of Pharmacy, Henan University, Kaifeng 475004, Henan, P. R. China

^9^Department of Oncology, the Affiliated Hospital of Southwest Medical University, Luzhou 646000, Sichuan, P. R China

**Results**


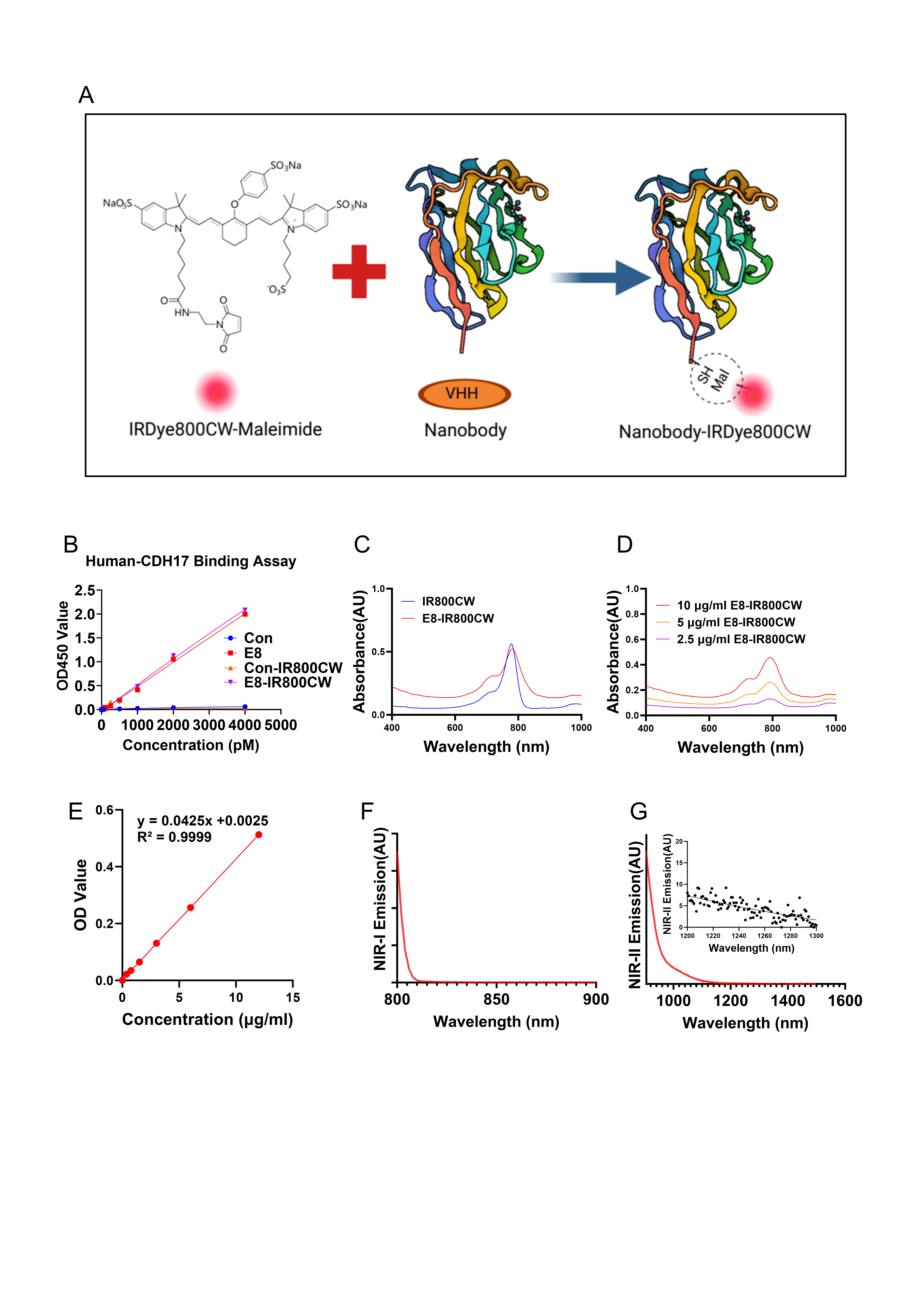


**Fig. S1.** (A) Schematic representation of the reaction of nanobodies with IR800CW. (B) ELISA assay to confirm the binding capability of E8 nanobody and E8-IR800CW to CDH17domin1-3 (*n* = 3). (C) UV–vis absorption spectra of free IR800CW and E8-IR800CW. (D) UV–vis absorption spectra of Absorption of different concentrations E8-IR800CW. (E) The OD778 value of E8-IR800CW displays a linear correlation with the concentration within a certain range. (F)and (G) Emission spectra in NIR-I/II spectra of E8-IR800CW measured at a concentration of 10 µg/ml (IR800CW).


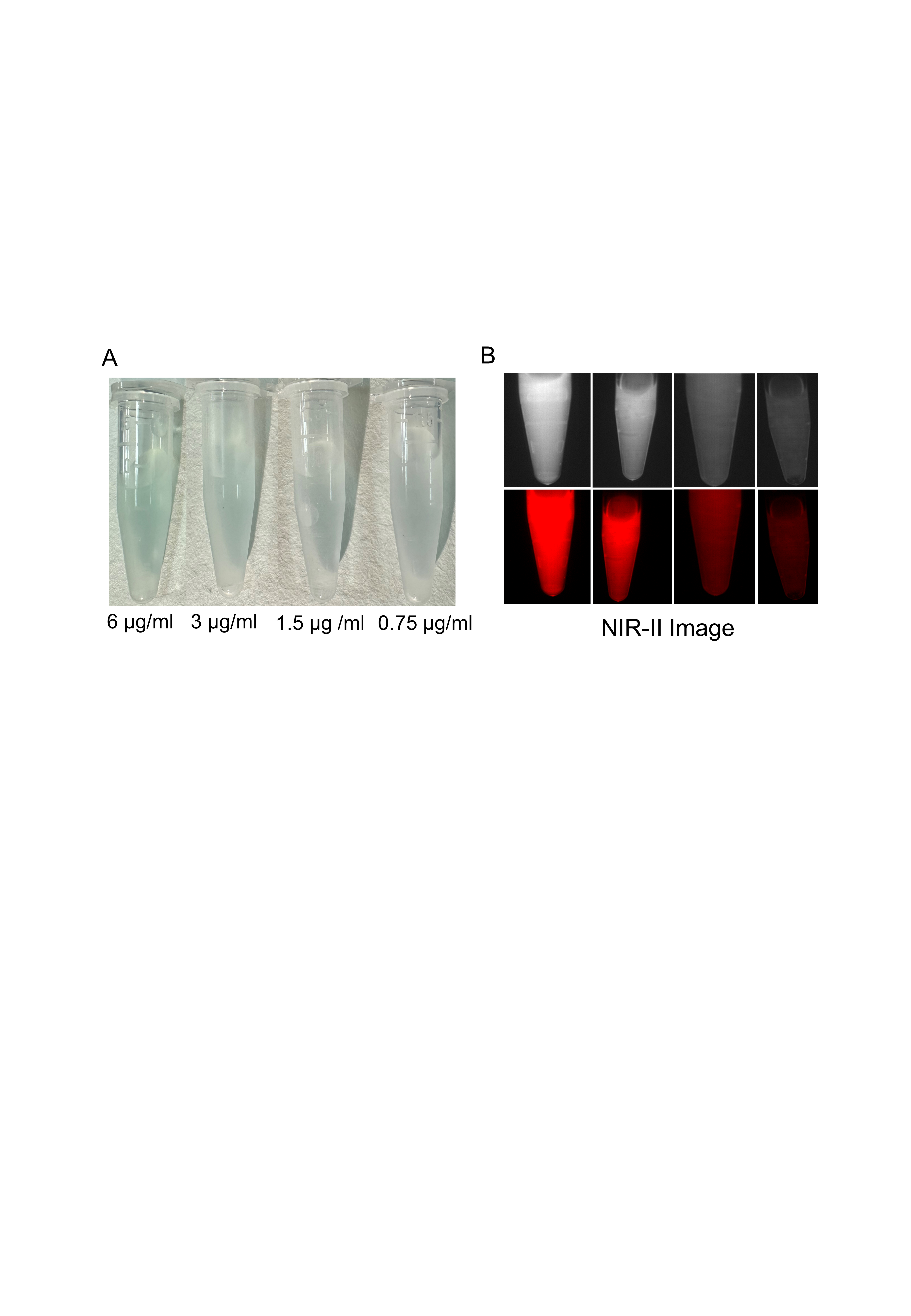


**Fig. S2.** (A) Images of different concentrations of E8-IR800CW under bright light. (B) Imaging results of different concentrations of E8-IR800CW under the NIR-II imaging system (Excitation wavelength 808nm, exposure time 100ms, filter 900LP). Up: NIR-II images; bottom: Pseudo-color fluorescence images.


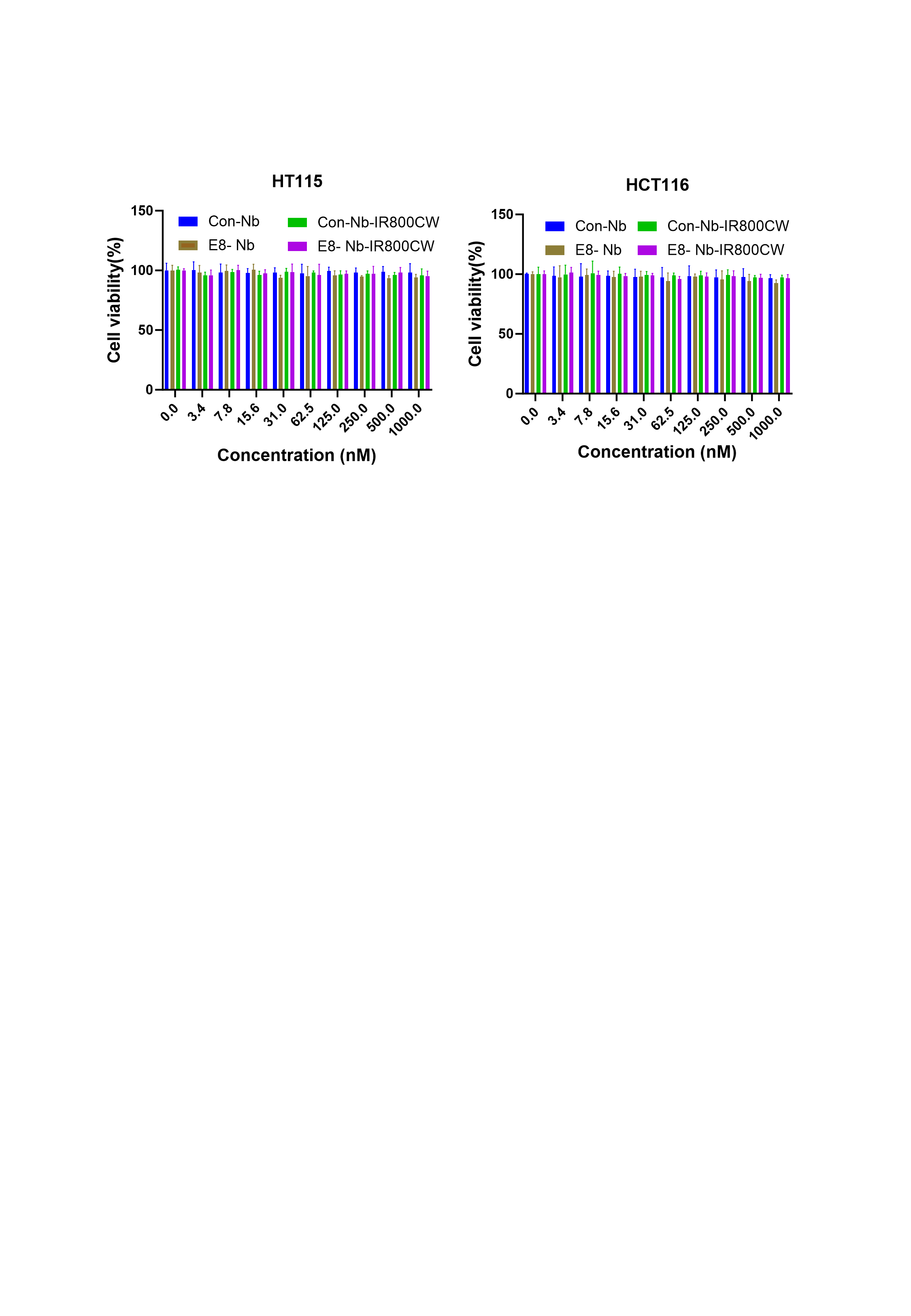


**Fig. S3.** Cell viability assay of CRC cell lines (HT115 and HCT116) treated with Con-Nb, E8-Nb, Con-Nb-IR800CW and E8-Nb-IR800CW (n=3).


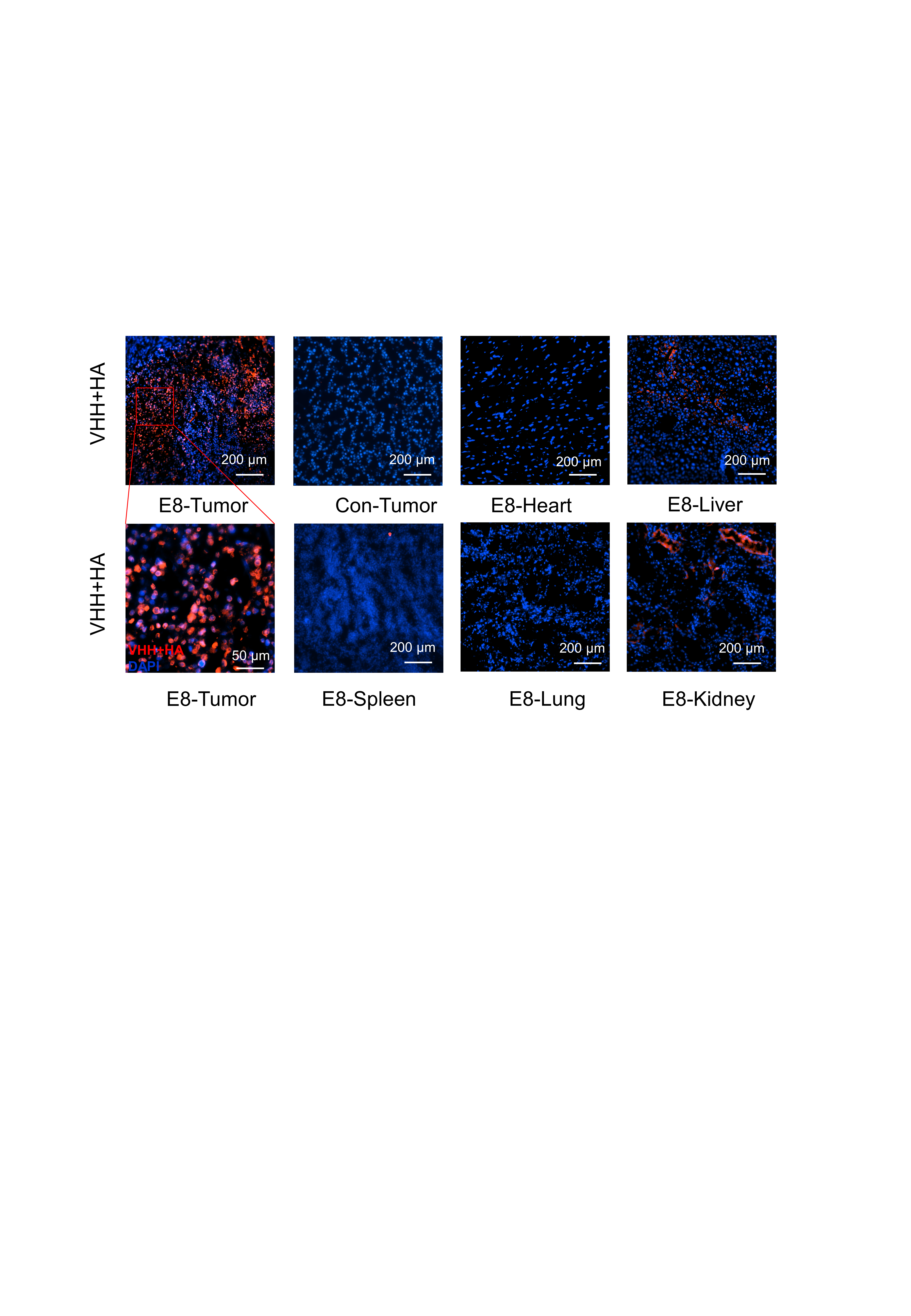
**Fig. S4.** Distribution of E8-IR800CW in tumor and control tissues related to Fig. 3A. Scale bar = 200 μm. Immunofluorescence staining was conducted with anti-VHH and anti-HA tag antibodies (red). E8 -IR800CW specifically accumulated in CDH17 positive tumor tissue, and liver and kidney tissues showed unspecific staining due to phagocytosis and excretion.


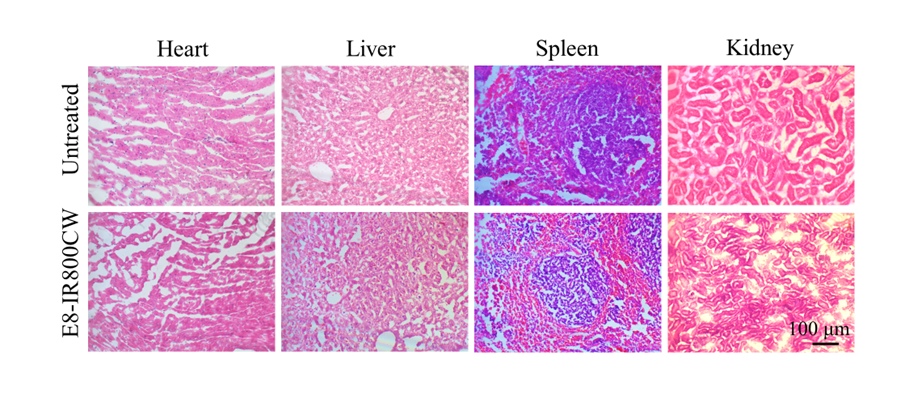


**Fig. S5.** H&E staining in frozen sections for major organs from untreated and E8-IR800CW treated mice. Untreated mice were used as a control. No pathological change was observed in the major organs from two groups (n=3). Scale bars = 100 µm.


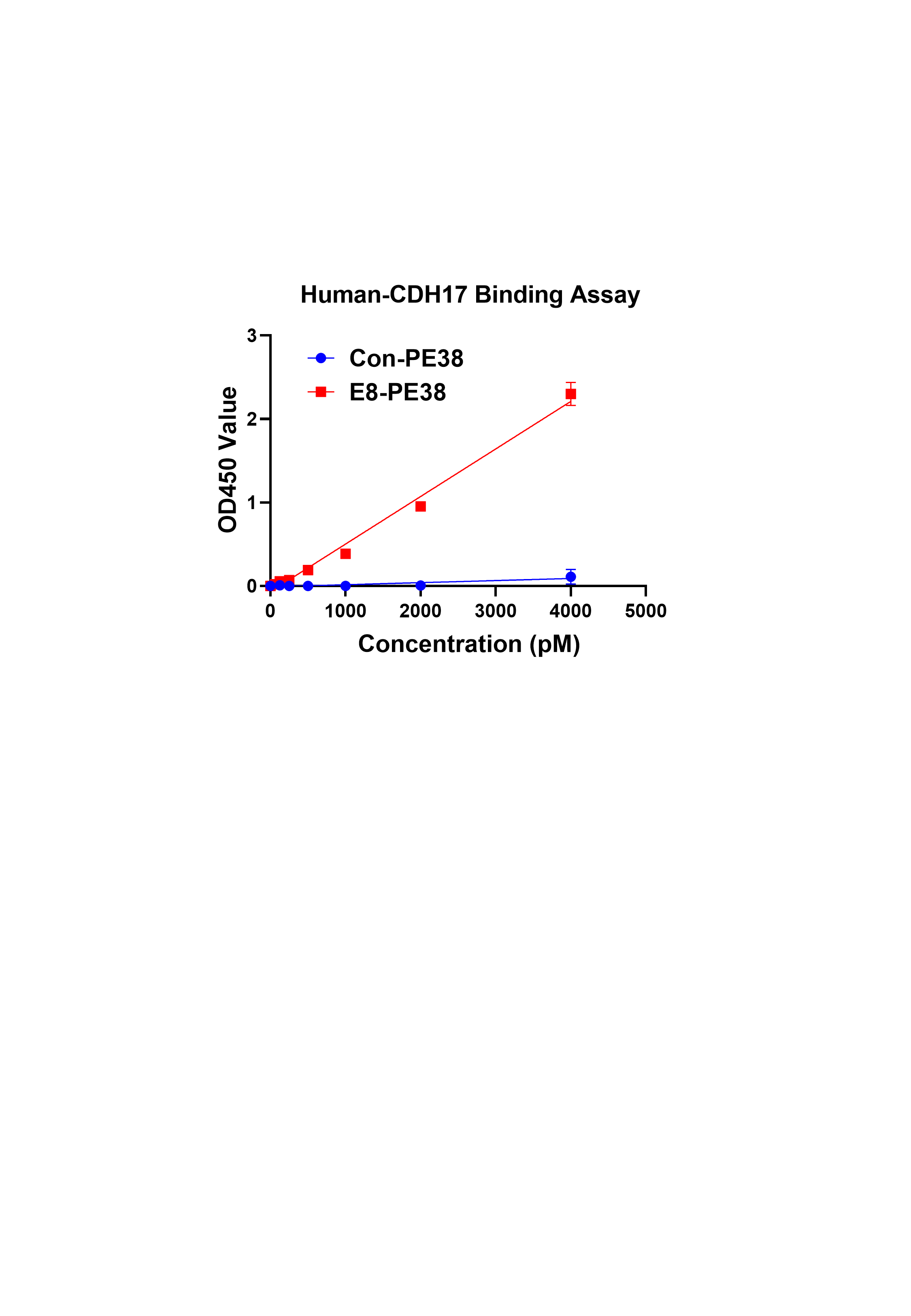


**Fig. S6.** ELISA assay to confirm the binding capability of E8-PE38 to CDH17 domain (*n* = 3); E8-PE38 exhibited strong antigen binding ability.


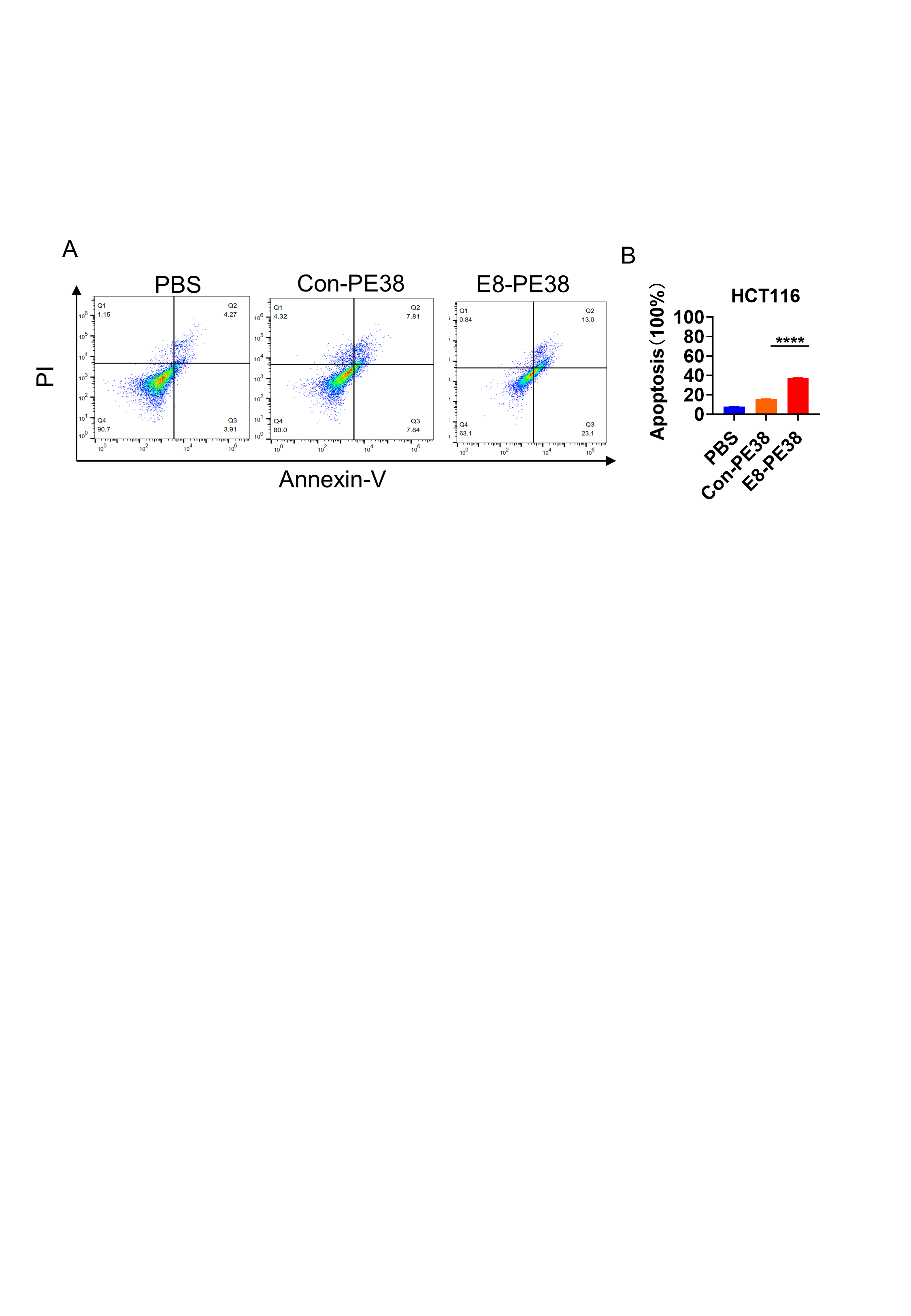


**Fig. S7.** (A) Detection of apoptotic cells in HCT116 cells with different groups by flow cytometry. (B) The quantitative results for Fig. S7A, showed that E8-PE38 promoted more significantly apoptosis than Con-PE38 or vehicle (n=3, *p* < 0.001).


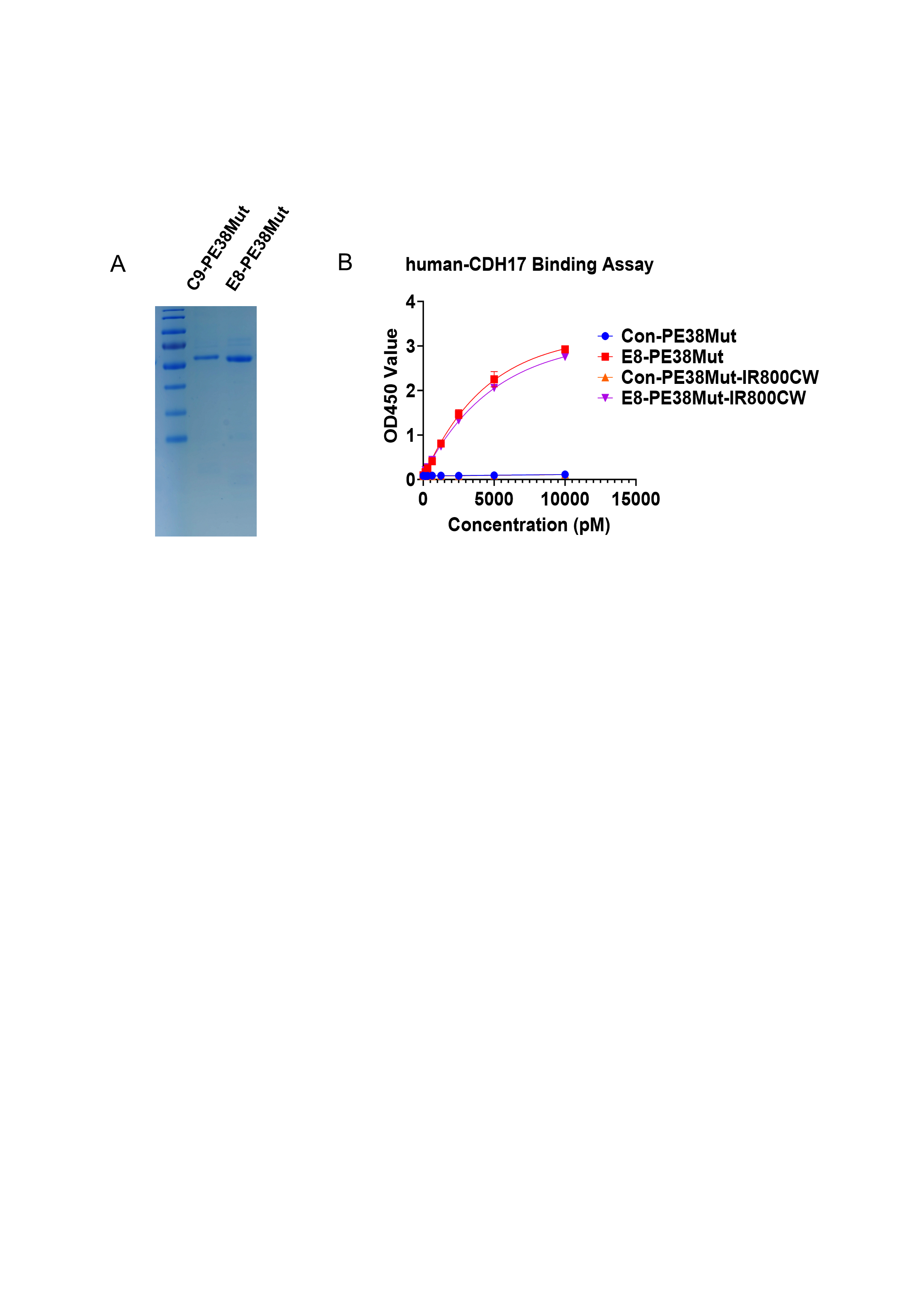


**Fig. S8.** (A) SDS-PAGE analysis of Nb-PE38Mut. (B) ELISA assay to confirm the binding capably of E8-PE38Mut and E8-PE38Mut-IR800CW to CDH17 protein (n = 3). E8-PE38Mut maintained the strong ability to bind CDH17 antigen after labelled with IR800CW.


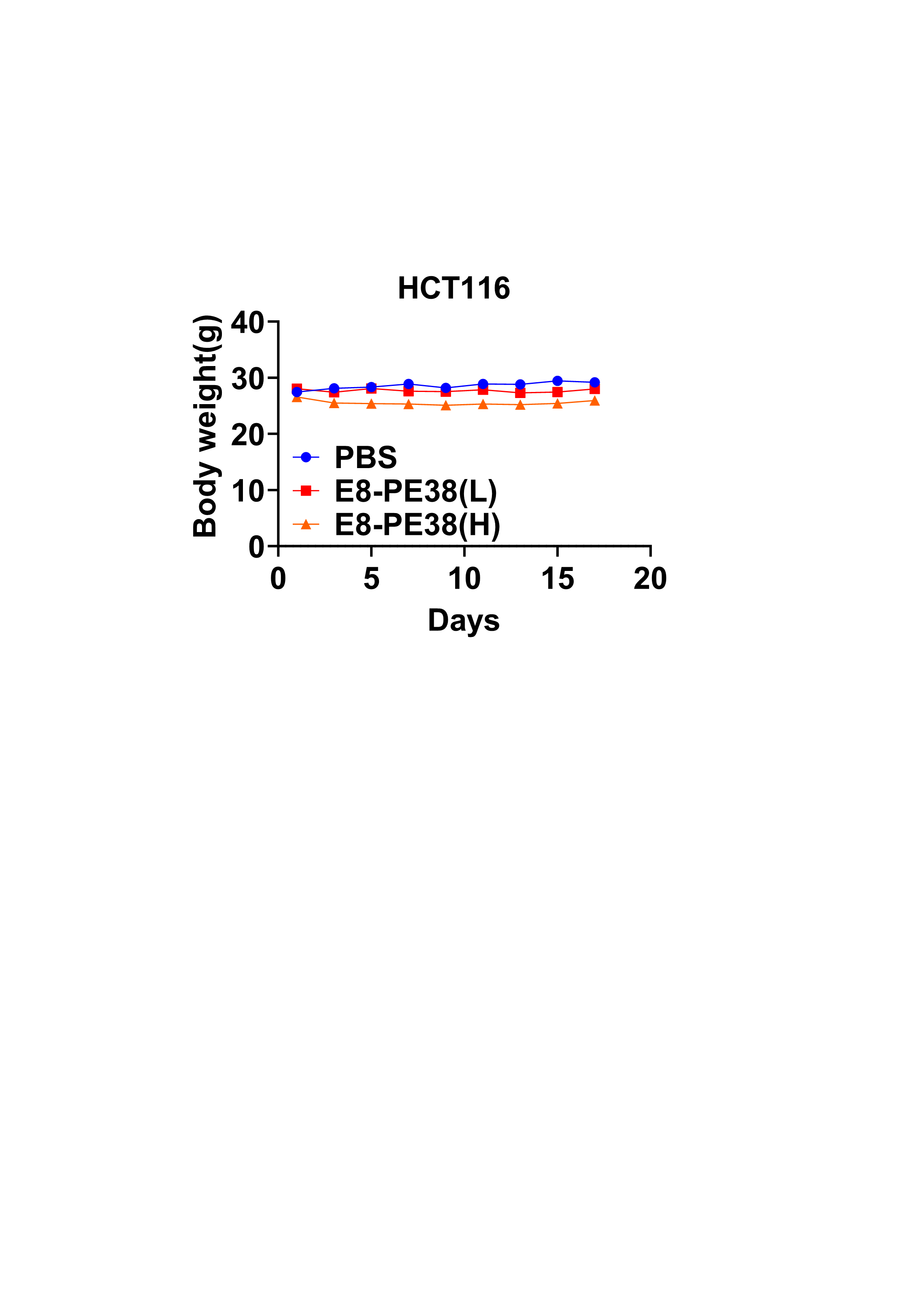


**Fig. S9.** Body weight of mice during the treatment from three groups shown in Fig. 6B.


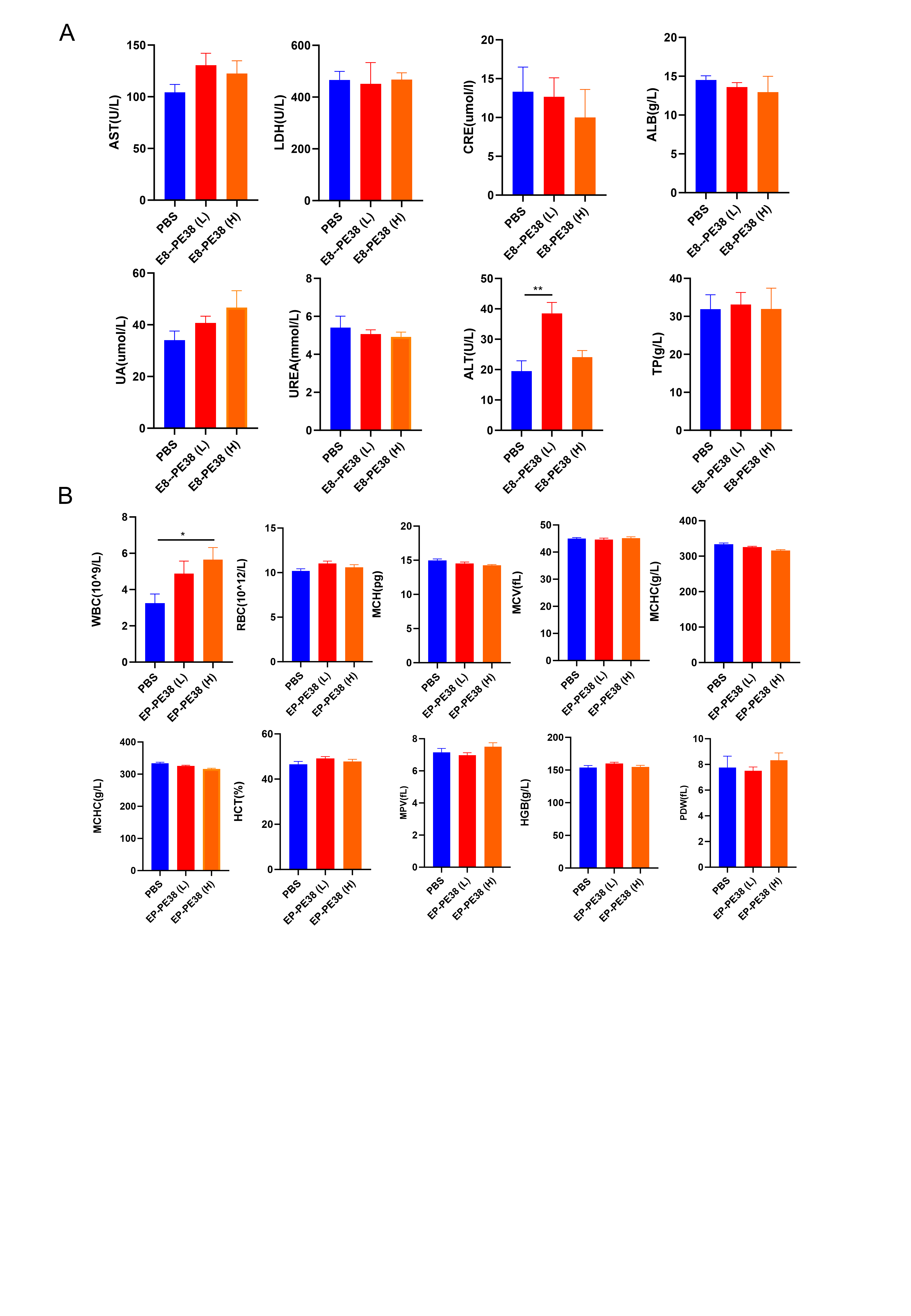


**Fig. S10.** The results of blood biochemistry and blood cell counting in treated mice related to Fig. 6B. (A) Blood biochemistry did not show any significant differences between the three groups receiving different treatments except ALT with a slight increase. (B) No significant changes were found in the various parameters of blood cell counts except WBC. WBC, white blood cell; RBC, red blood cell; HGB, hemoglobin; HCT, hematocrit; MCV, mean corpuscular volume; MCH, mean hemoglobin; MCHC, mean hemoglobin concentration; PDW, platelet distribution width; MPV, mean platelet volume; AST, aspartate aminotransferase; ALB. albumin; LDH, lactate dehydrogenase; ALT, alanine aminotransferase; CRE, creatinine; UA, uric acid; UREA, urea; TP, total serum protein.


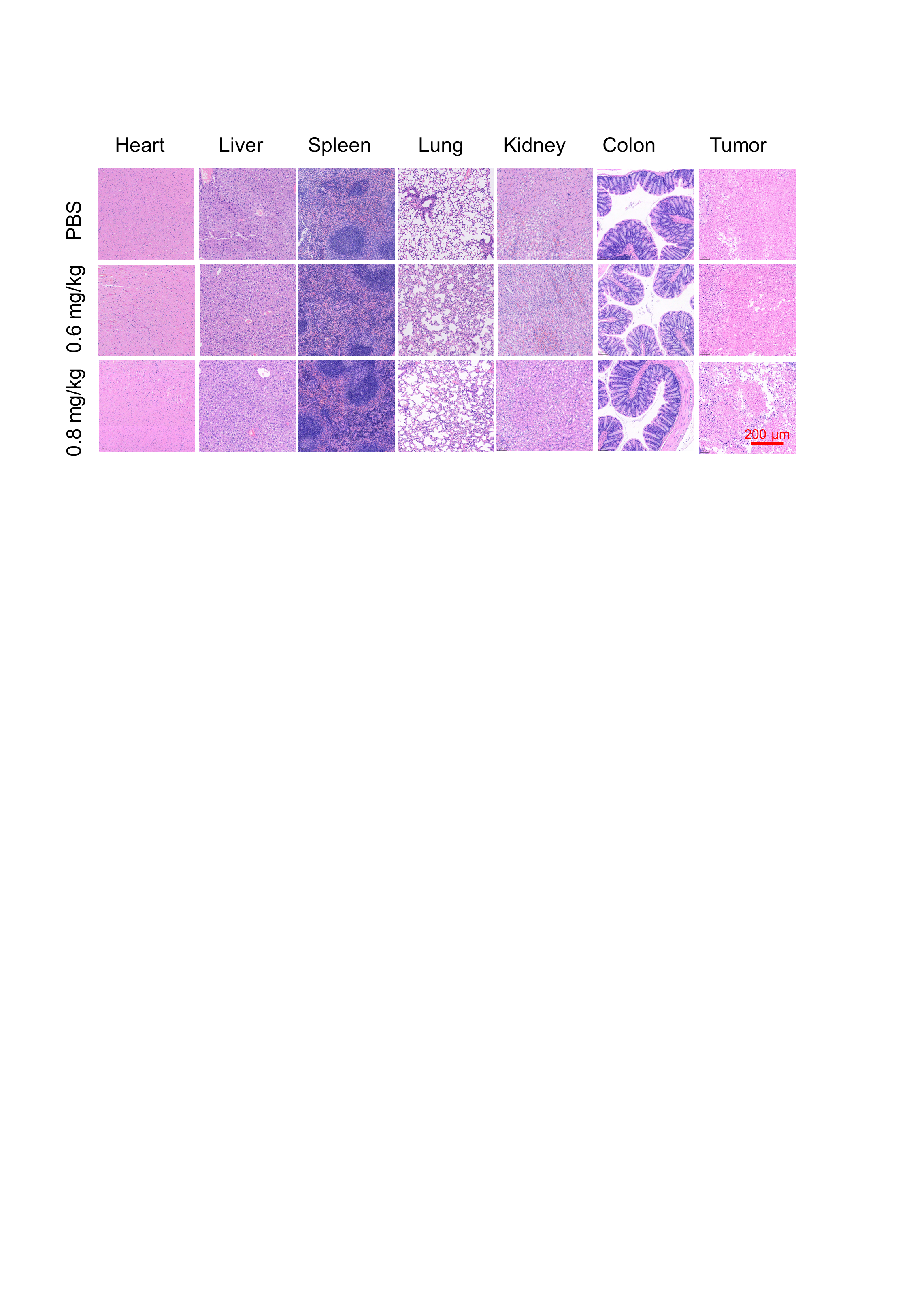


**Fig. S11.** H&E staining for major organs from mice treated in Fig. 6B. No pathological change was observed in the major organs from three groups. Scale bars = 200 μm.


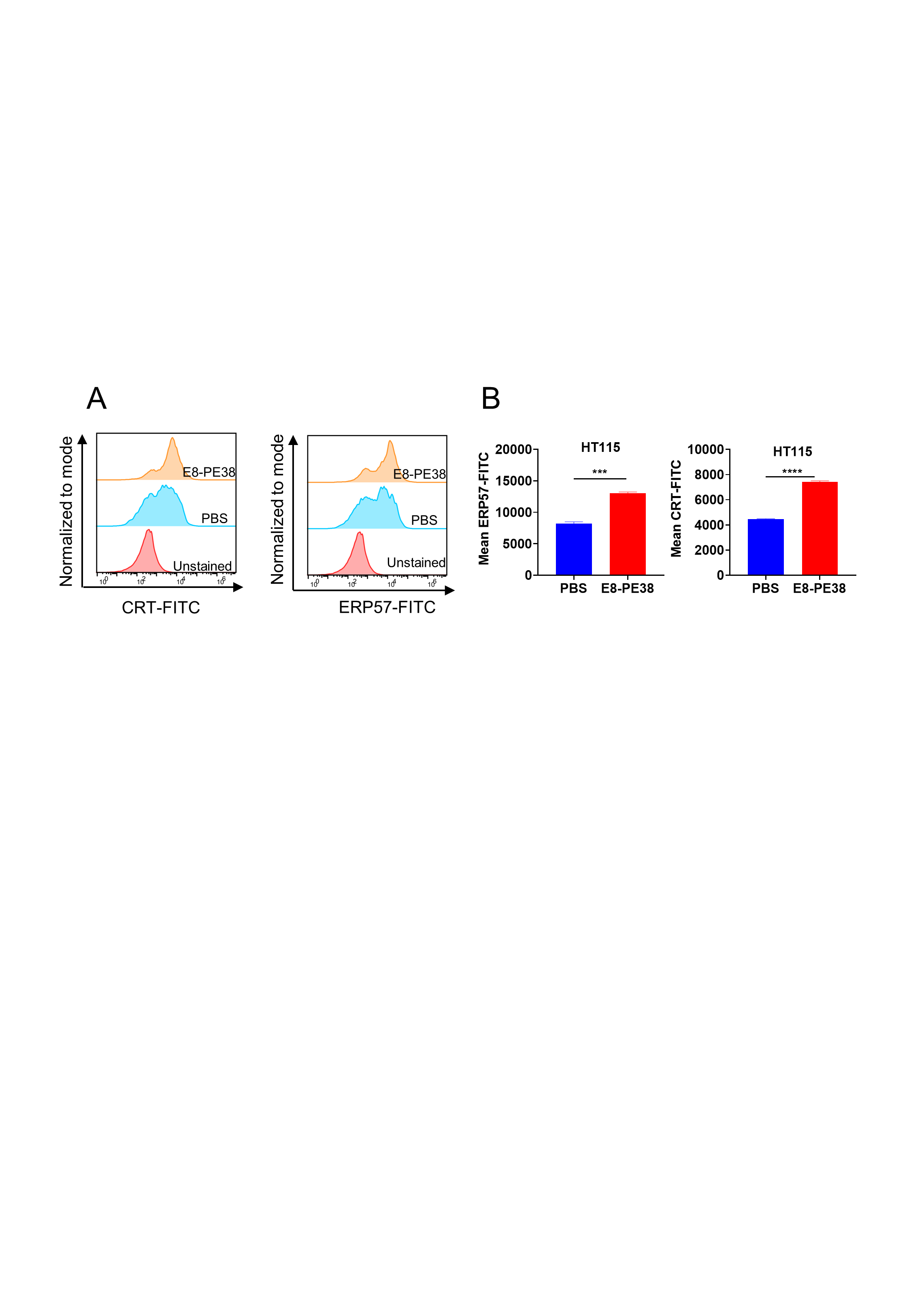


**Fig. S12.** (A) The detection of CRT and ERp57 expression on cell membranes with flow cytometry after E8-PE38 treatment in HT115 cells. (B) The quantitation for CRT and ERp57 expression in Fig. S12A.


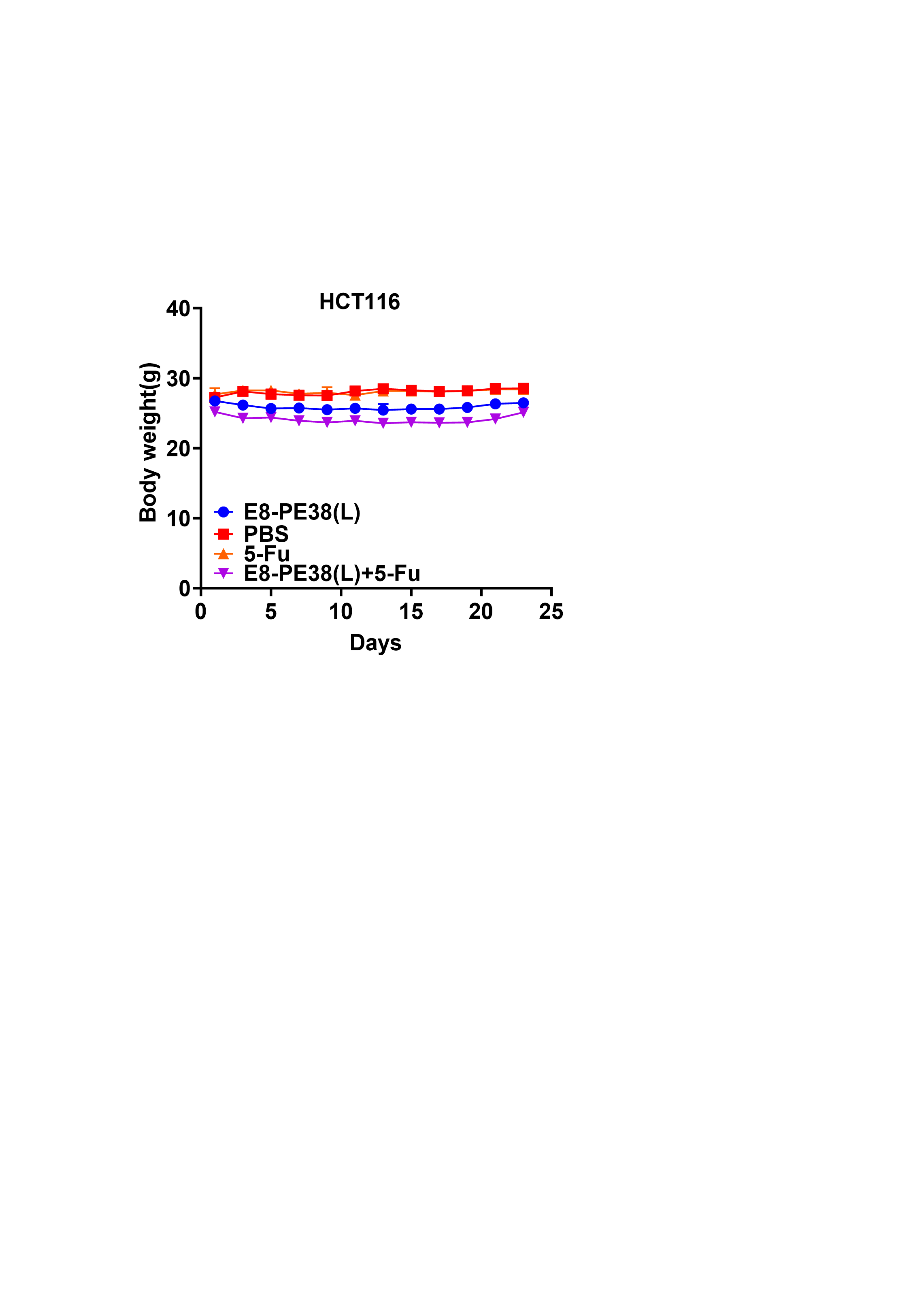


**Fig. S13.** Body weight during the treatment from four groups shown in Fig. 7L.

**Materials and Methods:**

**ELISA**

To verify the binding activity of E8-Nb to the CDH17 domain 1-3 protein, ELISA was conducted in the following procedures: 10 µg/ml recombinant human CDH17 domain 1-3 protein was coated onto 96-well plates and incubated overnight at 4°C. The uncoated protein was washed with PBS. After blocking with 3% BSA (Bovine serum albumin, BSA), the plate was incubated with nanobodies of different concentrations in PBST (PBS with 0.1% Tween 20) solution for 1 hour at RT. The plate was washed three times with PBST and again incubated for 1 hour at RT with anti-HA-HRP (1:3000). Afterwards, the plate was washed three times with PBST again, and 100 µl of tetramethylbenzidine (Solarbio, China) was added to each well. The reaction was stopped at the appropriate time point, and the optical density was measured at 450 nm in a microplate reader.

**Cell ELISA**

To verify the binding ability of the fluorescent probe (E8-IR800CW) to CRC cell lines, CRC cells were cultured at a cell density of 3 x 10^4^ per well in 96-well ELISA plates (CORING Cat# 3603). After 24 hours, the cells were washed with ice-cold PBS, fixed with 2% PFA for 10 minutes, and then washed with ice-cold PBS solution again. Subsequently, cells were incubated with a 4% donkey serum solution at RT for 1 hour. Different concentrations of the fluorescent probe (E8/Con-IR800CW) were added and incubated at RT for another 1 hour. The plates were subsequently washed three times with PBST, and the fluorescence intensity was measured under a 784 nm laser channel by a Sapphire Capture system (Sapphire, USA).

***In vitro* cell viability assay**

To validate the ability of E8-PE38 immunotoxin to inhibit the proliferation of CRC cells, CRC cells were seeded in 96-well cell culture plates at 4× 10^3^ cells/well. After 24 hours, the three purified protein solutions (E8, E8-PE38, and Con-PE38) were added at different concentrations (0, 1.95, 3.91, 7.8125, 15.625, 31.25, 62.5, 125, 250, 500, and 1000 nM). After incubation for 72 hours, 10μl of CCK-8 (**Cell Counting Kit-8, CCK-8**) solution (MCE, China) was carefully added to each well and incubated for 1 hour. Absorbance was then measured at 450 nm by an automated microplate reader (LabServ K3 TOUCH, Thermo Fisher Scientific, USA). Data for cell viability and IC50 (half maximal inhibitory concentration, IC50) values were analyzed using GraphPad Prism.

***In vitro* cellular uptake of** **nanobodies and immunotoxins**

The 6-well plate was seeded with 3 x 10^5^ cells and incubated overnight. Nanobody and immunotoxin, labeled with Cy5 fluorescent dye, were added to the cells. After incubation for 1 hour or 4 hours, the cells were gently washed three times with ice-cold PBS to remove unbound nanobodies or immunotoxins. Then, the cells were digested and the nanobody and immunotoxin bound to the cell surface were removed with 0.25% trypsin for 5 min, and the cells were fixed with 2% PFA for 10 min and washed with ice-cold PBS. The fluorescence intensity of the cells was observed by flow cytometry.

**Flow cytometry**

For CDH17 surface staining: Various cells were gently washed twice with precooled PBS, followed by digestion with 0.25% EDTA (Ethylenediaminetetraacetic acid, EDTA). The cells were then fixed with 0.25% fresh PFA for 5 min at RT and blocked with 3% BSA. Afterward, the CDH17 antibody recognizing both human and mouse CDH17 was mixed with the cells on ice for 1 hour, and the cells were then washed with PBS to remove residual CDH17 antibody. The cells were next incubated with a secondary antibody (anti-rabbit Alexa 488) in the dark for 1 hour. Finally, the cells were washed three times with PBS and analyzed using Beckman Flow Cytometry and Flow Jo software.

For apoptosis flow cytometry analysis: The 6-well plate was used, and CRC cells were seeded with a density of 2.0 x 10^5^ cells/well. After overnight incubation, the E8-PE38 and Con-PE38 were then diluted with fresh RIPM1640 with 2% FBS and incubated with cells for another 72 hours. The cells were washed with cold PBS and then digested with trypsin. The cells were then stained with 500 μl of binding buffer containing FITC-Annexin-V and PI and analyzed using Beckman Flow Cytometry and Flow Jo software. The untreated cells were used as a negative control.)

**Western blot**

Various CRC cells were lysed in RIPA lysis buffer containing protease inhibitor cocktail and phosphatase inhibitor cocktail, and the protein concentrations were determined by a BCA protein assay kit. The protein samples, mixed with 5x loading buffer (Beyotime, China), were boiled for 10 minutes. Next, 20 μg of total protein was loaded onto a 10% SDS‒PAGE (Sodium dodecyl sulfate-polyacrylamide gel electrophoresis, SDS-PAGE) gel before conducting electrophoresis. The proteins were transferred to PVDF (Polyvinylidene fluoride, PVDF) membranes with Wet/Tank Blotting Systems (Bio-Rad, USA). Subsequently, the PVDF membranes were blocked with 5% skim milk in TBST (1×TBS(Tris buffered saline, TBS) with 0.1% Tween 20) for 2 h, and were further incubated at 4°C overnight with the following antibodies: anti-CDH17 antibody (Abcam Cat: A5286), anti-Caspase3/p17/p19 antibody (Proteintech Cat: 19677-1-AP), anti-Bcl2 polyclonal antibody (Proteintech Cat: 26593-1-AP), anti-Bax antibody (Abcam Cat: ab32503), anti-phospho-eIF2α (Cell Signaling Technology Cat: 9721S), anti-β-Actin antibody (Cell Signaling Technology Cat: 3700S), and anti-ATF4 antibody (Proteintech Cat: 10835-AP). After incubation, the membranes were washed five times in TBST and then incubated with rabbit or mouse secondary antibody conjugated with HRP for 1 hour at RT. Finally, the target proteins were detected by enhanced chemiluminescence. Quantitative analysis of target protein gray values was analyzed by ImageJ software.

**Immunofluorescence staining**

For cellular immunofluorescence, CRC cells were seeded with the density of 4 x 10^4^ cells/well in a 24-well plate with coverslips and incubated overnight. Before fixation with 0.25% PFA, the cells were washed three times using PBS. The solution containing anti-CDH17 antibody was added after blocking with 3% BSA. The cells were washed with PBS, and Alexa Fluor 594-conjugated anti-rabbit antibody was used to amplify the signals. After 1-h incubation at RT, the cells were washed three times with PBS, and DAPI (4',6-Diamidino-2-Phenylindole, DAPI) was added for nuclear staining for 10 min. Then, the cells were washed three times with PBS and photographed with a fluorescence microscope.

For tissue immunofluorescence, tissues were dehydrated with a sucrose solution at 4°C overnight. Subsequently, tissues were embedded with O.C.T. (optimal cutting temperature, OCT) Compound (Sakura Finetek, USA) and stored at -80°C until use. The tissues were sectioned into 10 μm slices. They were then fixed with acetone at -20°C for 10 minutes and washed twice with TBS. Subsequent procedures were similar to those used for cellular immunofluorescence.

**Protein purification**

Various recombinant plasmids including pET-14B-E8-Nb, pET-14B-Con-Nb, pET-14B-E8-Nb-PE38, pET-14B-Con-Nb-PE38, pET-14B-E8-Nb-PE38 mut and pET-14B-Con-Nb-PE38 mut were transformed into BL21 (DE3), and then the bacterial clones were incubated at 37 °C and 220 rpm in LB broth containing ampicillin until OD600 value reached 0.6, followed by protein induction with 0.2 mM IPTG (Isopropyl β-D-Thioacetamide, IPTG) at 16 °C and 220 rpm overnight. The bacterial cultures were then centrifuged at 4000 rpm for 30 minutes at 4°C. The bacterial precipitates were dissolved in lysis buffer (300 mM NaCl, 50 mM NaH_2_PO_4_, 10 mM imidazole, pH 8.0, 1 mM PMSF (Phenylmethanesulfonylfluoride)), followed by three rounds of high-pressure crushing in the lysis buffer at 4°C. The lysates were centrifuged at 12,000 rpm for 30 minutes, and the supernatants were loaded onto a gravity column containing 1.5 mL of Ni-NTA agarose resin (Smart-lifesciences, China) and incubated at 4°C for 1 hour. non-specific proteins bound to resins were removed with 50 ml of washing buffer I (300 mM NaCl, 50 mM NaH_2_PO_4_, 20 mM imidazole, pH 8.0, 1 mM PMSF) and 50 ml of washing buffer II (300 mM NaCl, 50 mM NaH_2_PO_4_, 50 mM imidazole, pH 8. 0, 1 mM PMSF), followed by 15 ml of elution buffer (300 mM NaCl, 50 mM NaH_2_PO_4_, 250 mM imidazole, pH 8.0, 1 mM PMSF) to elute the target proteins. The protein solutions were desalted by Amicon Ultra-15 Centrifugal Filters (Millipore) and were buffer exchanged into PBS to remove imidazole. The purified proteins were identified by SDS-PAGE gel and western blotting with His or HA tag antibody. The proteins with 10% glycerol were aliquoted and frozen in liquid nitrogen and stored at -80 ℃ until use.
